# ADAM10 and ADAM17 regulate EGFR, c-Met and TNF RI signalling in liver regeneration and fibrosis

**DOI:** 10.1038/s41598-021-90716-3

**Published:** 2021-06-01

**Authors:** Olga Zbodakova, Karel Chalupsky, Lenka Sarnova, Petr Kasparek, Marketa Jirouskova, Martin Gregor, Radislav Sedlacek

**Affiliations:** 1 Laboratory of Transgenic Models of Diseases, Institute of Molecular Genetics of the Czech Academy of Sciences, Průmyslova 595, 252 50 Vestec, Czech Republic; 2 Czech Centre of Phenogenomics, Institute of Molecular Genetics of the Czech Academy of Sciences, Průmyslova 595, 252 50 Vestec, Czech Republic; 3 Laboratory of Integrative Biology, Institute of Molecular Genetics of the Czech Academy of Sciences, Vídeňská 1083, 142 20 Prague, Czech Republic

**Keywords:** Proteolysis, Functional genomics, Liver diseases

## Abstract

ADAM10 and ADAM17 are proteases that affect multiple signalling pathways by releasing molecules from the cell surface. As their substrate specificities partially overlaps, we investigated their concurrent role in liver regeneration and fibrosis, using three liver-specific deficient mouse lines: ADAM10- and ADAM17-deficient lines, and a line deficient for both proteases. In the model of partial hepatectomy, double deficient mice exhibited decreased AKT phosphorylation, decreased release of EGFR activating factors and lower shedding of HGF receptor c-Met. Thus, simultaneous ablation of ADAM10 and ADAM17 resulted in inhibited EGFR signalling, while HGF/c-Met signalling pathway was enhanced. In contrast, antagonistic effects of ADAM10 and ADAM17 were observed in the model of chronic CCl_4_ intoxication. While ADAM10-deficient mice develop more severe fibrosis manifested by high ALT, AST, ALP and higher collagen deposition, combined deficiency of ADAM10 and ADAM17 surprisingly results in comparable degree of liver damage as in control littermates. Therefore, ADAM17 deficiency is not protective in fibrosis development per se, but can ameliorate the damaging effect of ADAM10 deficiency on liver fibrosis development. Furthermore, we show that while ablation of ADAM17 resulted in decreased shedding of TNF RI, ADAM10 deficiency leads to increased levels of soluble TNF RI in serum. In conclusion, hepatocyte-derived ADAM10 and ADAM17 are important regulators of growth receptor signalling and TNF RI release, and pathological roles of these proteases are dependent on the cellular context.

## Introduction

The liver is an organ with remarkable regenerative capacity. Yet, once this ability is compromised, liver transplantation often remains the only solution. Therefore, studying mechanisms contributing to the maintenance of its homeostasis is important. In the widely used rodent model of liver regeneration, 2/3 partial hepatectomy, two thirds of the liver are excised which induces hepatocyte proliferation in the remaining tissue to compensate for the original liver mass^1,2^. In the initial phase of regeneration after partial hepatectomy, differentiated G_0_ hepatocytes are stimulated by cytokines tumor necrosis factor alpha (TNF-α) and interleukin 6 (IL-6)^3,4^ and re-enter cell cycle. In the following phase, in which hepatocytes proceed through cell cycle, two growth receptor pathways play a crucial part: epidermal growth factor receptor (EGFR)^5,6^ and hepatocyte growth factor (HGF) receptor (c-Met) pathway^7–9^. Combined elimination of both EGFR and HGF/c-Met pathways fatally disrupted homeostasis in an unchallenged liver^10^ and completely hindered liver regeneration after partial hepatectomy^11^.

EGFR can be activated by different ligands: epidermal growth factor (EGF), transforming growth factor α (TGFα), amphiregulin (AREG), betacellulin, heparin-binding EGF-like growth factor (HB-EGF), epiregulin and epigen^12^. In the liver, a major source of AREG and TGFα are the hepatocytes themselves^13^, HB-EGF is predominantly produced by non-parenchymal liver cells^14^, and EGF is supplied to the liver from intestinal cells^15^. In contrast to the EGFR pathway, the signalling pathway downstream of the cMet receptor is activated exclusively by HGF^16,17^, which is mainly produced by non-parenchymal liver cells^18^. Ligand binding leads to phosphorylation of growth receptors and activation of downstream mediators (such as ERK1/2, STAT3 and AKT)^2^.

Several of the mentioned protein factors critical for liver regeneration are released from the cell surface by proteases. The most important are the membrane-bound zinc-binding proteases ADAM17 and ADAM10, whose activation leads to substrate cleavage proximal to the cell surface^19^, in the process called ectodomain ‘shedding’. Although whole body deficiency of ADAM10 as well as ADAM17 results in lethality in mice^20,21^, liver development was not compromised. Still, ADAM10 seems to be an important regulator for liver homeostasis maintenance as liver-specific ablation of ADAM10 resulted in spontaneous fibrogenesis, characterized by unbalanced differentiation of hepatocytes and deregulated expression of the bile acid transporters^22^.

ADAM17 is an important regulator of TNF-α pathway. Besides cleavage of TNF-α itself, which is necessary in order to bind and activate its receptor, ADAM17 also sheds TNF receptor 1 (TNF RI) and TNF receptor 2 (TNF RII)^23^, which leads to pathway inactivation. ADAM17 activity thus regulates TNF-α signalling in two directions and the outcome of ADAM17 activity on TNFα signalling therefore depends on the cellular context. EGFR pathway is regulated by ADAMs^24,25^ through the release of EGFR ligands. Though EGFR signalling defects in mouse models were mainly associated with ADAM17 deficiency^26,27^, in vitro data show that both ADAM10 and ADAM17 are able to cleave some of the EGFR substrates^24,28^. Liver-specific ADAM17-deficient mice exhibited significantly reduced AREG levels, but hepatocyte proliferation after partial hepatectomy was not affected in these mice^29^. This was quite surprising as AREG-deficient mice showed delayed proliferation of hepatocytes after partial hepatectomy^16^. It is possible that other EGFR substrates compensated the lack of AREG in ADAM17-deficient mice. Indeed, in vitro study by Le Gall et al.^30^ showed that ADAM10 can release substrates primarily shed by ADAM17, such as TGF-α or HB-EGF, in conditions when ADAM17 is missing. Similar to the EGFR pathway, HGF signalling is also regulated via the shedding process. Several studies have shown that the HGF receptor c-Met is cleaved by both ADAM10 and ADAM17^28,31,32^.

In conclusion, ADAM10 and ADAM17 are implicated in several pathways crucial for liver regeneration. In this work, we aimed to examine the combined role of ADAM10 and ADAM17 in liver regeneration and liver fibrosis development, using mouse models deficient in ADAM10, ADAM17 or simultaneously deficient for both proteases.

## Results

### Generation of liver-specific ADAM10- and ADAM17-deficient mouse models

**Figure 1:**
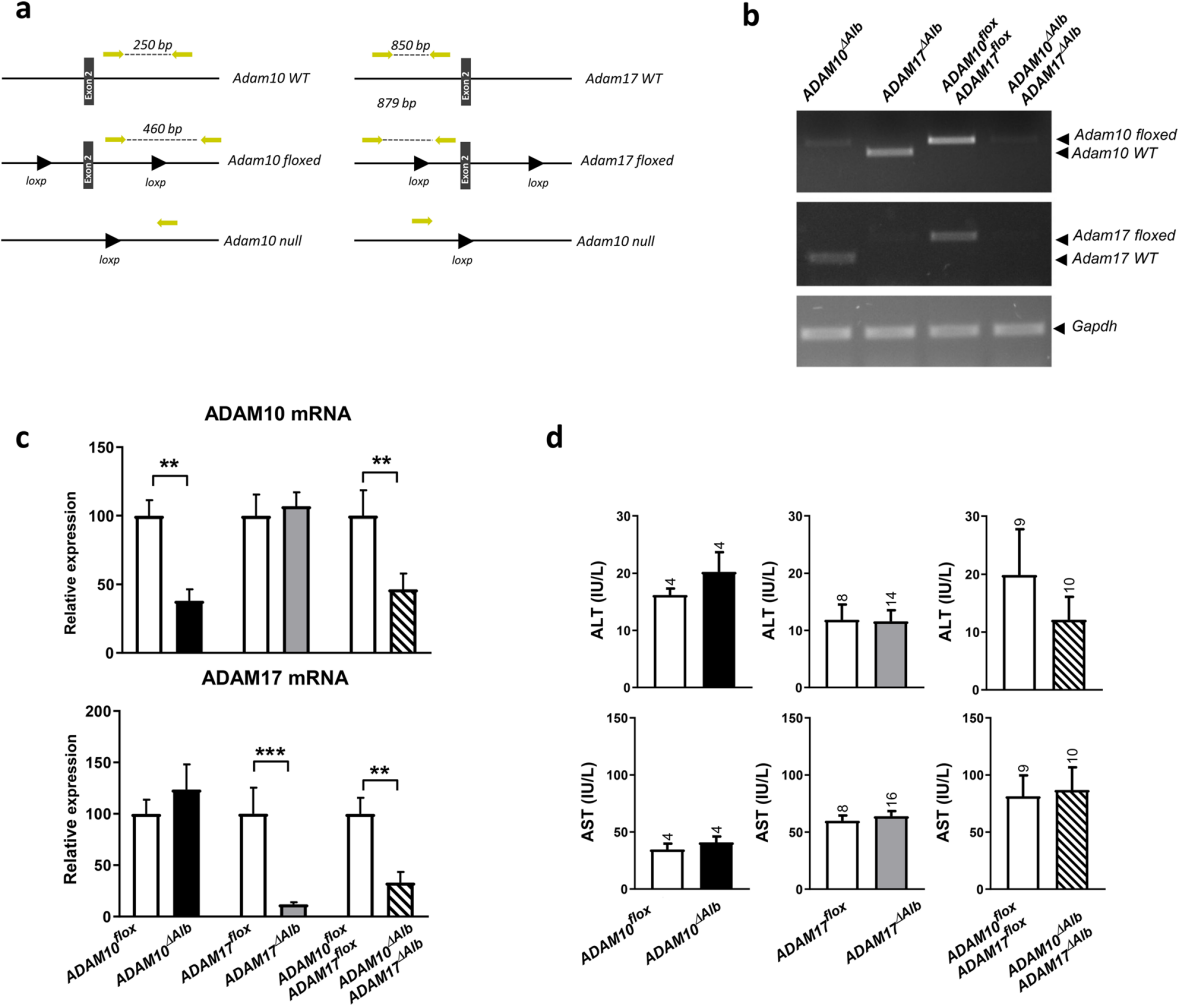
Alb-Cre mediated deletion of ADAM10 and ADAM17 genes. (**a**) Diagram of a genotyping strategy depicting sizes of the PCR bands in respective alleles. (**b**) Semiquantitative PCR showed almost complete elimination of floxed exons in genomic DNA of primary hepatocytes isolated from ADAM10^ΔAlb^, ADAM17^ΔAlb^ and ADAM10^ΔAlb^ADAM17^ΔAlb^ mice. Cre negative littermates from ADAM10^ΔAlb^ADAM17^ΔAlb^ were used as controls. (**c**) Quantitative RT-PCR showed significant reduction of *Adam10* and *Adam17* mRNA levels in whole liver tissue of ADAM10^ΔAlb^, ADAM17^ΔAlb^ and ADAM10^ΔAlb^ADAM17^ΔAlb^ mice. Expression was normalized to *Gapdh*. (n = 5 per group, mean ± SEM, **p < 0.01. Significance was determined by analysis of ΔCt.) (**d**) ALT and AST levels in serum from unchallenged 32 weeks old mice showed no hepatocellular damage in either of studied lines. (n per group is indicated as a number above the bar, mean ± SEM).

To study the role of ADAM10 and ADAM17 in hepatic injury, we generated three liver-specific deficient lines, using a mouse line expressing Cre under albumin promoter. Besides single deficient ADAM10^ΔAlb^ and ADAM17^ΔAlb^ lines, we also generated a double deficient ADAM10^ΔAlb^ADAM17^ΔAlb^ line as both proteases share several substrates, which has been previously described as important in liver pathology. The ablation of *Adam10* and *Adam17* genes was verified by semiquantitative PCR using genomic DNA isolated from primary hepatocytes (Fig. 1a,b). Nonetheless, we cannot exclude that a tiny portion of hepatocytes still express ADAM10 or ADAM17 proteins as a weak bands detecting WT alleles were present in the PCR from DNA of the deficient hepatocytes. Decrease in Adam10 and Adam17 expression was confirmed by quantitative PCR using RNA isolated from whole liver tissue (Fig. 1c).

Previously Muller et al.^22^ reported that ADAM10-deficient mice, which had stronger expression of Cre driven by α-fetoprotein promotor, but same tissue specificity as our ADAM10^ΔAlb^, develop spontaneous fibrosis. Interestingly, in our experimental setup low serum activity of alanine aminotransferase (ALT) and aspartate aminotransferase (AST), which are the markers of hepatocellular damage, indicated that neither ADAM10^ΔAlb^, nor ADAM17^ΔAlb^, ADAM10^ΔAlb^ADAM17^ΔAlb^ mice developed spontaneous liver damage (Fig. 1d). Furthermore, the gross morphology of liver did not display any abnormalities in any of the mutant lines.

### ADAM10^ΔAlb^ADAM17^ΔAlb^ mice have reduced AKT activation after partial hepatectomy

**Figure 2:**
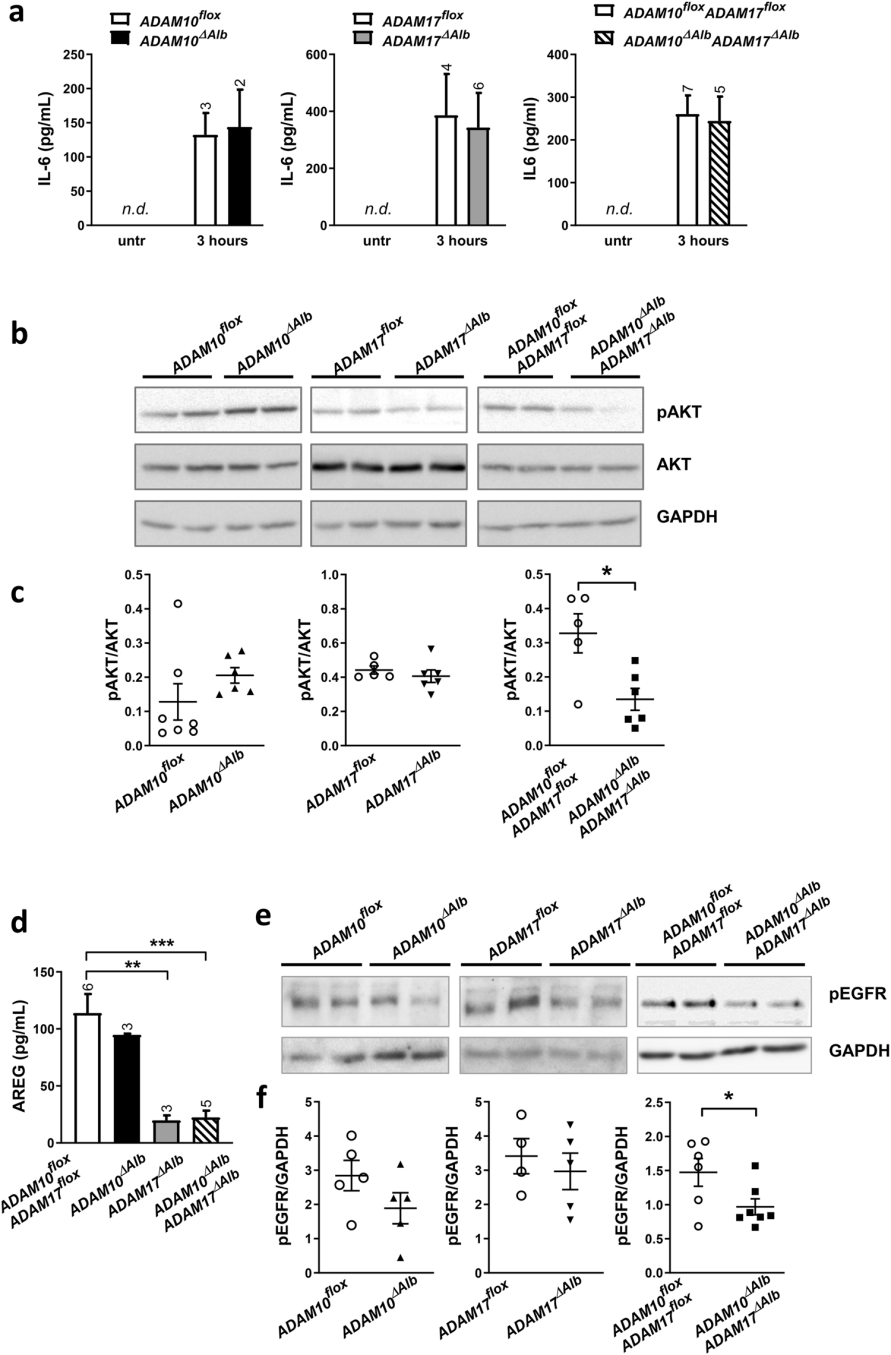
Analysis of pro-survival pathways in the priming phase of liver regeneration. (**a**) Elevated levels of IL-6 determined by ELISA in serum from mice 3 h post hepatectomy were observed in all studied groups. IL-6 was not detected in untreated (Untr) mice. (n per group is indicated as a number above the bar in each time point, mean ± SEM) (**b**) phospho-AKT (pAKT) and AKT in liver lysates from animals 6 h post hepatectomy was evaluated by immunoblotting. ADAM10^ΔAlb^ADAM17^ΔAlb^ exhibited significantly lesser extent of AKT phosphorylation than controls littermates. (**c**) Densitometric analysis of immunoblot from (**b**) (*p < 0.05). (**d**) Analysis of culture media by ELISA determined reduced shedding of AREG from surface of ADAM17^ΔAlb^ and ADAM10^ΔAlb^ADAM17^ΔAlb^ primary hepatocytes compared to controls. (n indicated above the bar, mean ± SEM , **p < 0.01, ***p < 0.001 (**e**) Control primary hepatocytes were stimulated for 30 min with sera taken from mice 6 h post hepatectomy. Cell lysates analysed by immunoblotting showed that mouse serum from ADAM10ΔAlbADAM17ΔAlb mice has reduced ability to induce phosphorylation of EGFR compared to serum from control littermates.) (**f**) Densitometry of immunoblot from (**e**) (*p < 0.05).

As unchallenged animals (ADAM10^ΔAlb^, ADAM17^ΔAlb^, ADAM10^ΔAlb^ADAM17^ΔAlb^) did not display any significant alteration in markers of liver damage when compared to their control littermates, we tested whether ADAM10/17 deficiency had an effect on model liver pathologies. First, we examined the regenerative ability of the liver by applying the model of 2/3 partial hepatectomy. The typical pattern of elevated levels of cytokine IL-6, corresponding to activation of resident liver macrophages, was observed in the serum of all treated animals three hours post hepatectomy with no difference between ADAM10^ΔAlb^, ADAM17^ΔAlb^, ADAM10^ΔAlb^ADAM17^ΔAlb^ mice and control littermates (Fig. 2a). To study the activation of pro-survival signals following the initial priming, we analysed the activation of AKT kinase, which plays a crucial role in proliferation of hepatocytes^33^. Immunoblot analysis of whole liver lysates of hepatectomized mice revealed comparable levels of AKT phosphorylation in ADAM10^ΔAlb^ and ADAM17^ΔAlb^ mice compared to control littermates 6 h post hepatectomy (Fig. 2b,c). In contrast, double deficient ADAM10^ΔAlb^ADAM17^ΔAlb^ mice exhibited significantly reduced levels of phosphorylated AKT at this time point. This suggests that both ADAM10 and ADAM17 contribute to the release of factors which trigger proliferative signals after partial hepatectomy and that they are interchangeable in activation of AKT pathway.

### Reduced amounts of EGFR activating factors in serum of ADAM10^ΔAlb^ADAM17^ΔAlb^ mice 6 h after partial hepatectomy

As the EGFR pathway is a well-established target of both ADAM10 and ADAM17, we next analysed whether reduced AKT activation might result from aberrant EGFR signalling. Therefore, we first checked the extent of release of AREG, a well-known EGFR ligand, in primary hepatocyte cultures. AREG levels in culture media were measured by ELISA at 6 h in cultures of untreated ADAM10^ΔAlb^ADAM17^ΔAlb^ and control hepatocytes in the presence of 10% FBS. ADAM10^ΔAlb^ADAM17^ΔAlb^ and ADAM17^ΔAlb^ primary hepatocytes exhibited a dramatically reduced ability to effectively shed AREG (Fig. 2d), while shedding from ADAM10^ΔAlb^ hepatocytes did not significantly differ from the controls.

Compromised AREG shedding in ADAM10^ΔAlb^ADAM17^ΔAlb^ hepatocytes prompted us to check the release of EGFR ligands in vivo—in serum from peripheral blood of mice after partial hepatectomy. However, AREG is only one of the several EGFR ligands, therefore we designed the experiment in a way to determine overall potential to activate EGFR, analysing the combined effect of all different EGFR ligands. We incubated control hepatocytes for 30 min in the presence of serum obtained from ADAM10^ΔAlb^, ADAM17^ΔAlb^ and ADAM10^ΔAlb^ADAM17^ΔAlb^ mice or control littermates 6 h post hepatectomy and evaluated its ability to trigger EGFR phosphorylation. Immunoblot analysis revealed that serum from ADAM10^ΔAlb^ADAM17^ΔAlb^ triggered significantly lower phosphorylation of EGFR than serum from controls, while ADAM10^ΔAlb^ and ADAM17^ΔAlb^ serum was comparable to serum from controls (Fig. 2e,f). This result strongly suggests that the shedding of EGFR ligands is compromised in ADAM10^ΔAlb^ADAM17^ΔAlb^ mice at the beginning of the regeneration process in the liver.

### ADAM10^ΔAlb^ADAM17^ΔAlb^ hepatocytes show no defect in proliferation in response to partial hepatectomy

Lower levels of AKT phosphorylation in ADAM10^ΔAlb^ADAM17^ΔAlb^ at early time points after hepatectomy suggested a slower proliferative response; therefore, we next examined hepatocyte proliferation in the following stages of liver regeneration.

**Figure 3:**
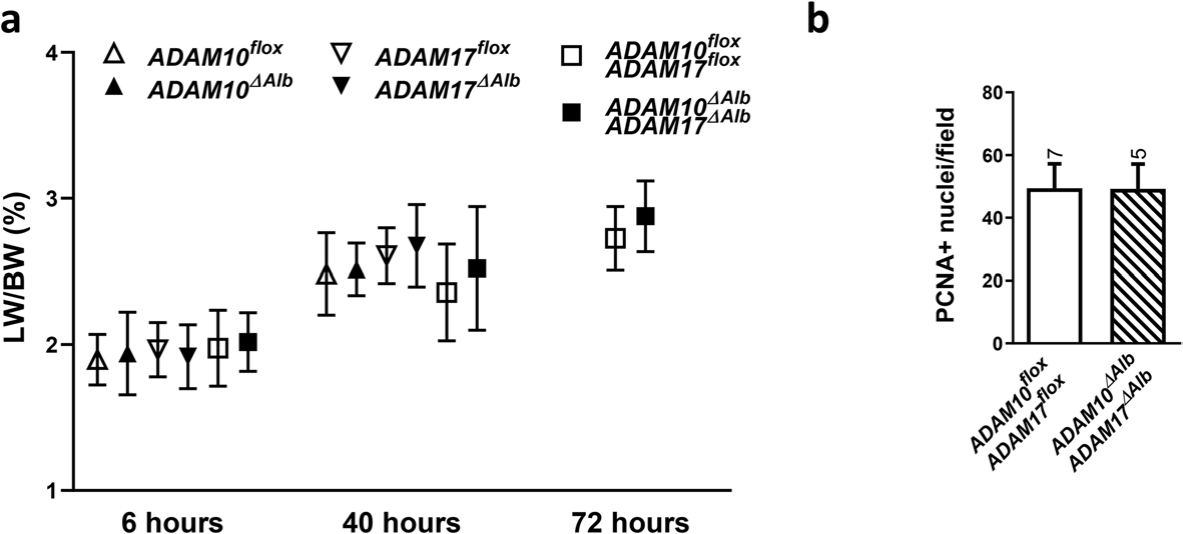
Proliferation after partial hepatectomy. (**a**) Gain of relative liver weight was monitored 6, 40 and 72 h post hepatectomy. No differences in liver weight to body weight ratio (LW/BW) between groups were observed. (mean ± SD, n = 8–10 mice for each line per time point) (**b**) Immunohistochemical staining of liver sections revealed no difference in number of PCNA positive (PCNA +) between ADAM10^ΔAlb^ADAM17^ΔAlb^ and control littermates 40 h post hepatectomy. (n indicated above the bars, mean ± SEM).

To follow liver mass restoration, we monitored changes in liver weight to body weight ratio mice consecutively up to three days after hepatectomy. In this time frame, ADAM10^ΔAlb^ADAM17^ΔAlb^ mice gained liver mass to comparable extent as controls, reaching increases of 50% within 72 h (Fig. 3a). ADAM10^ΔAlb^ and ADAM17^ΔAlb^ were monitored until 40 h after hepatectomy and no significant differences were detected. To examine whether the described increase reflected the rate of hepatocyte division, we next analysed hepatocytes proliferation using immunohistological staining of proliferating cell nuclear antigen (PCNA) (Fig. 3b). For quantification, only nuclei corresponding to hepatocytes, identified by their typical round shape, were counted, while all non-parenchymal cells were excluded from the analysis. Surprisingly, no difference in number of PCNA positive nuclei was detected between ADAM10^ΔAlb^ADAM17^ΔAlb^ and control mice at 40 h post hepatectomy (Fig. 3b). Similarly, PCNA staining in ADAM10^ΔAlb^ indicated no difference (Fig. S1). This suggests that despite the initial decrease in the AKT activation, likely caused by decrease in EGFR ligands release, ADAM10^ΔAlb^ADAM17^ΔAlb^ mice are able to proceed with hepatocyte proliferation as effectively as their control littermates.

### HGF triggers stronger signalling through cMet in ADAM10^ΔAlb^ADAM17^ΔAlb^ hepatocytes

**Figure 4:**
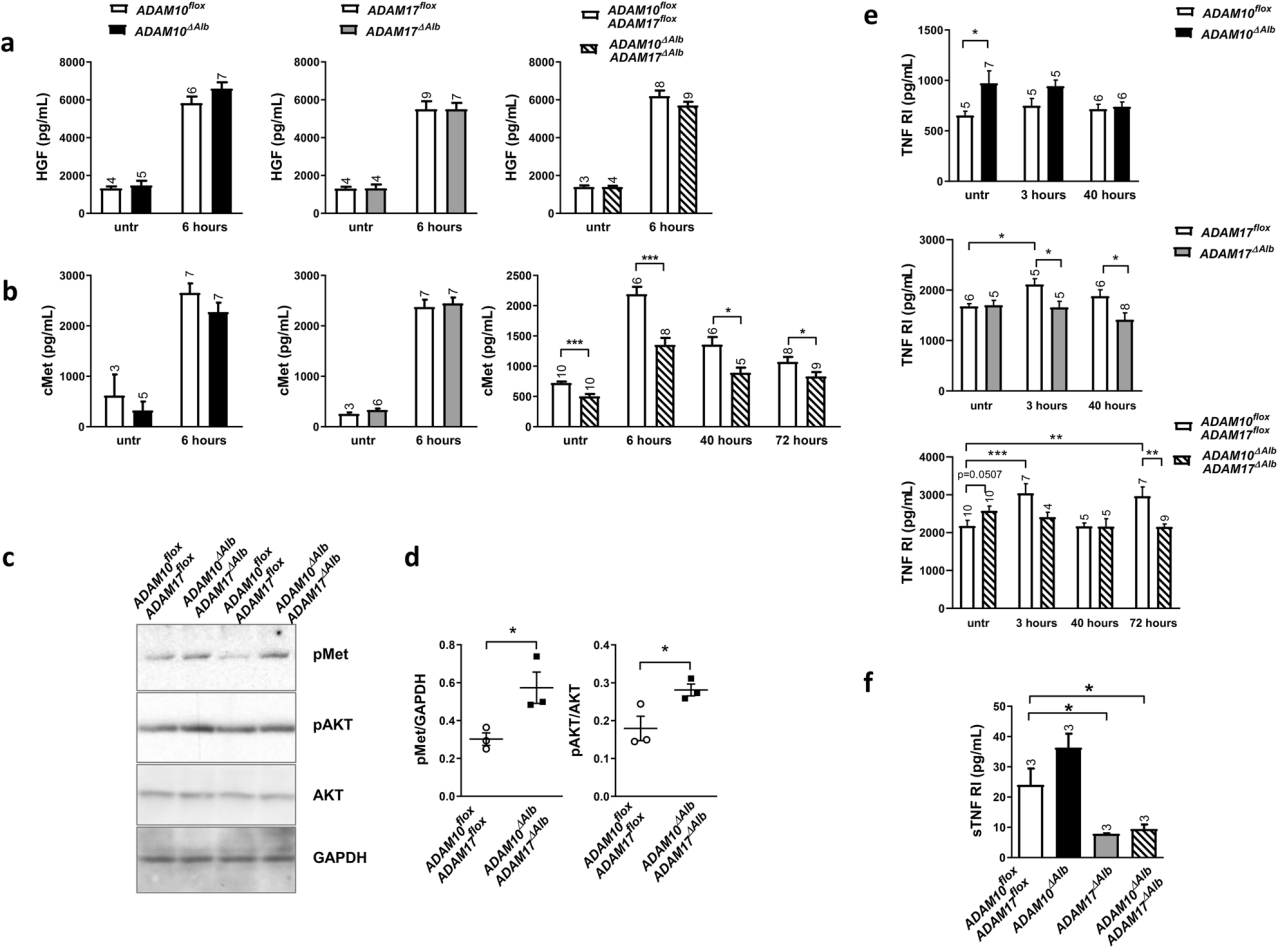
HGF and TNF RI signalling in ADAM10^ΔAlb^ADAM17^ΔAlb^ after hepatectomy. (**a**) ELISA analysis of mice serum display elevated levels of HGF 6 h post hepatectomy in all studied groups of mice. (n indicated above the bars, mean ± SEM) (**b**) Shedding of c-Met into serum increases after hepatectomy. Levels of shed c-Met, determined by ELISA, were lower in ADAM10^ΔAlb^ADAM17^ΔAlb^ in all studied time points post hepatectomy. (n indicated above the bars, mean ± SEM, *p < 0.05, ***p < 0.001) (**c**) Phosphorylation of c-Met receptor (pMet) and AKT in primary hepatocytes after 30 min HGF treatment was analysed in lysates by immunoblotting. ADAM10^ΔAlb^ADAM17^ΔAlb^ primary hepatocytes show higher phosphorylation of cMet and AKT than control primary hepatocytes. (**d**) Densitometric evaluation of immunoblots from (**c**). Representative of three independent experiments shown. (*p < 0.05). (e) Serum levels of sTNF RI, determined by ELISA, were increased after hepatectomy. sTNF RI levels are higher in ADAM10^ΔAlb^ mice, while ADAM17^ΔAlb^ and ADAM10^ΔAlb^ADAM17^ΔAlb^shed lower amounts of sTNF RI after hepatectomy. (n indicated above the bars, mean ± SEM, *p < 0.05, **p < 0.01, ***p < 0.001). (**f**) Levels of shed sTNF RI from primary hepatocytes into culture medium after 6 h of cultivation. (n indicated above the bars, mean ± SEM, *p < 0.05).

To explore the ability of ADAM10^ΔAlb^ADAM17^ΔAlb^ to compensate for lower EGFR activation, we examined other signalling pathways important for hepatocyte proliferation. The HGF/c-Met pathway was a prospective candidate, as it plays an important role in liver regeneration and c-Met is a substrate of both ADAM10 and ADAM17. To find out whether HGF/c-Met pathway is dysregulated in ADAM10^ΔAlb^ADAM17^ΔAlb^ mice in the model of partial hepatectomy, we next analysed serum levels of both HGF and the soluble form of the HGF receptor c-Met. ELISA analysis revealed a prominent increase of serum HGF levels in mice 6 h post hepatectomy, however no significant difference was observed in any of the studied mouse lines, ADAM10^ΔAlb^, ADAM17^ΔAlb^ or ADAM10^ΔAlb^ADAM17^ΔAlb^ compared to their control littermates (Fig. 4a). Together with increased levels of HGF, levels of c-Met were significantly elevated in control animals starting at 6 h post hepatectomy, remaining significantly above unchallenged levels at all studied time points (6, 40 and 72 h post hepatectomy, Fig. 4b). Similar situation was observed in ADAM10^ΔAlb^ and ADAM17^ΔAlb^ lines (Fig. 4b). However, we found that the level of soluble c-Met in serum of ADAM10^ΔAlb^ADAM17^ΔAlb^ was significantly reduced in comparison to control littermates at all studied time points (Fig. 4b).

Reduced c-Met levels in serum correspond to compromised shedding in ADAM10^ΔAlb^ADAM17^ΔAlb^ mouse and may imply a higher level of remaining functional c-Met on hepatocyte surface leading to amplified HGF signalling. To address this hypothesis in vitro, we isolated primary hepatocytes from ADAM10^ΔAlb^ADAM17^ΔAlb^ and control mice and treated them with HGF in serum free medium. Indeed, immunoblot analysis of hepatocyte lysates revealed higher levels of phosphorylation of both c-Met and AKT (Fig. 4c,d) in ADAM10^ΔAlb^ADAM17^ΔAlb^ compared to control hepatocytes upon HGF treatment. Thus, combined deficiency of ADAM10 and ADAM17 results in reduced shedding of c-Met, retention of a larger pool of unshed receptor on the hepatocyte surface and higher sensibility towards HGF.

### Shedding of TNF RI is affected by deficiency of ADAM17 and ADAM10 in unchallenged and hepatectomized mice

Based on previous genetic studies, TNF RI is one of the key negative regulators of hepatocyte proliferation^34^. As TNF RI is well-described substrate of ADAM17, we were interested in how ablation of ADAM17 and ADAM10 in hepatocytes affects its soluble levels (sTNF RI) in our models. In control mice, serum levels of sTNF RI were rapidly increased as soon as 3 h after hepatectomy (Fig. 4e, ADAM17^flox^ and ADAM10^flox^ADAM17^flox^). According to expectation, this increase was not observed in ADAM17^ΔAlb^ mice (Fig. 4e) and ADAM17^ΔAlb^ exhibited significantly decreased TNF RI levels 3 and 40 h post hepatectomy as compared to their control littermates. To our surprise, ADAM10^ΔAlb^ mice exhibited more extensive shedding of sTNF RI into serum than their control littermates (Fig. 4e). A situation in ADAM10^ΔAlb^ADAM17^ΔAlb^ mice resembled both ADAM10^ΔAlb^ and ADAM17^ΔAlb^ depending on the studied time point. Under physiological conditions, sTNF RI levels in ADAM10^ΔAlb^ADAM17^ΔAlb^ mice showed a tendency towards higher shedding (Fig. 4e, untr) similarly to ADAM10^ΔAlb^. After hepatectomy, the missing activity of ADAM17 caused a decrease in sTNF RI levels in ADAM10^ΔAlb^ADAM17^ΔAlb^ serum in comparison to ADAM10^flox^ADAM17^flox^ (Fig. 4e, 72 h). Overall, described differences in sTNF RI levels were not very extensive, but we must note that peripheral blood serum contains sTNF RI originating from all the different cell types of the body, while ADAMs were ablated only in liver parenchyma cells. The significance of those differences in the liver is most likely much stronger than observed from peripheral blood. Evaluating the shedding of sTNF RI from primary hepatocytes, we observed the same tendencies as in serum from mice and the inhibitory effect of ADAM17 is much more prominent (Fig. 4f). ADAM10^ΔAlb^ADAM17^ΔAlb^ primary hepatocytes exhibit inhibited shedding of TNF RI, comparable to ADAM17^ΔAlb^. As isolation of primary hepatocytes is linked to their activation due to the treatment with digestive enzymes, we can consider the state in primary hepatocytes closer to the conditions in hepatectomized rather than the unchallenged liver.

### ADAM10^ΔAlb^ mice exhibit higher susceptibility to CCl_4_-induced fibrosis

As we identified alterations in signalling pathways during liver regeneration in ADAM10^ΔAlb^ADAM17^ΔAlb^ mice, we were further interested how the deficiency of ADAM10 and ADAM17 may affect the development of liver injury. We therefore applied a model of 4-week CCl_4_-intoxication, which results in liver fibrosis.

**Figure 5:**
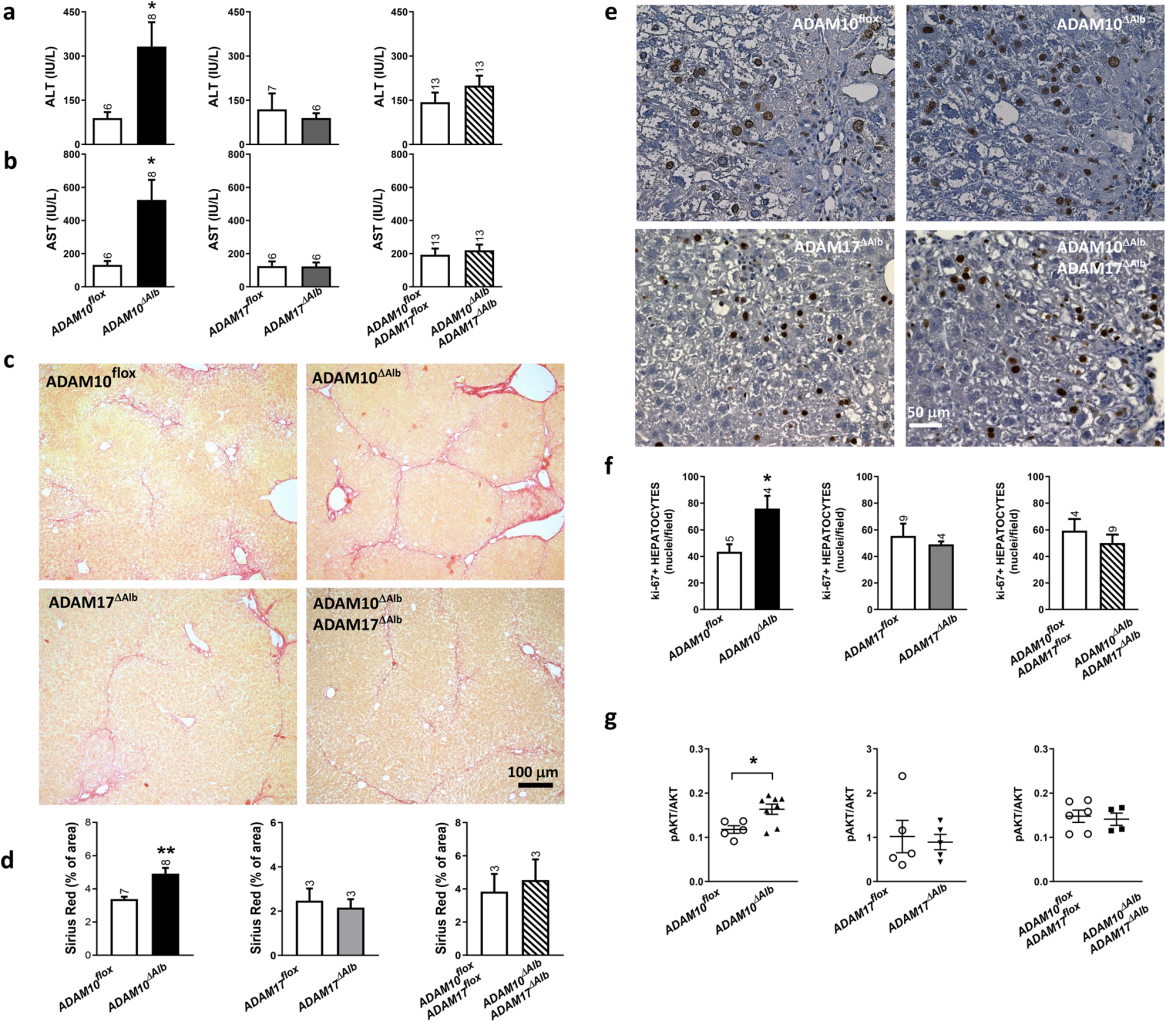
Model of fibrogenesis induced by CCl_4_ intoxication. (**a**,**b**) Serum levels of ALT and AST from mice after 4-weeks treatment with CCl_4_ show highest hepatocyte damage in ADAM10^ΔAlb^ mice. (n indicated above the bars, mean ± SEM,*p < 0.05) (**c**) Sirius red staining shows higher deposition of fibrotic collagens in ADAM10^ΔAlb^ than other studied groups. (**d**) Quantitative analysis of Sirius red-positive area. (n indicated above the bars, mean ± SEM, **p < 0.01) (**e**) Ki-67 staining shows than number of proliferating hepatocytes was highest in ADAM10^ΔAlb^ after 4-weeks treatment with CCl_4_. (**f**) Quantitative analysis of Ki-67 staining. Only nuclei corresponding to hepatocytes (identified by shape) were counted. (n indicated above the bars, mean ± SEM, *p < 0.05) (**g**) Immunoblotting of pAKT and total AKT in liver lysates revealed higher activation of AKT pathway in ADAM10^ΔAlb^ mice after CCl_4_ treatment. (*p < 0.05).

As expected, markers of liver injury (serum activity of alanine aminotransferase (ALT) and aspartate aminotransferase (AST)), were elevated in all studied groups after 4 weeks of CCl_4_ treatment. Strikingly the highest hepatocellular damage was found in ADAM10^ΔAlb^ animals (Fig. 5a,b), while ADAM17^ΔAlb^ mice exhibited the same extent of damage as their control littermates. Interestingly, serum aminotransferases were not increased in ADAM10^ΔAlb^ADAM17^ΔAlb^ in contrast to single ADAM10^ΔAlb^ mice. We further evaluated the development of fibrosis in liver by histopathological staining of fibrillary collagens, where the Sirius Red-positive area was determined by image analysis. Our results showed that higher levels of serum liver damage markers correlated with most extensive deposition of fibrillary collagens in ADAM10^ΔAlb^ mice when compared to other deficient lines and controls (Fig. 5c,d). Sirius Red staining (Fig. 5c) showed that at this time point ADAM10^ΔAlb^ developed a more advanced bridging phase of fibrosis, in which collagen scars already interconnected central veins. The extent of collagen deposits of ADAM17^ΔAlb^ and also ADAM10^ΔAlb^ADAM17^ΔAlb^ were comparable to controls. Fibrotic scars in these lines stretched from the central vein but were not prominent enough to form bridges. Moreover, Ki-67 staining revealed significantly higher hepatocyte proliferation (Fig. 5e,f) in the ADAM10^ΔAlb^ mouse line, which is most likely caused by a proliferative response to more severe damage caused by CCl_4_. In line with this finding, immunoblot analysis of pro-proliferative AKT pathway from liver lysates revealed higher phosphorylation of AKT in ADAM10^ΔAlb^ mice, which was not detected in ADAM17^ΔAlb^ or ADAM10^ΔAlb^ADAM17^ΔAlb^ mice (Fig. 5g).

A prior study^22^ has shown that ADAM10 downregulation decreased expression of several bile acid transporters, including Mrp2. As other members of Mrp family, Mrp3 and Mrp4, are involved in detoxification of CCl_4_, we analysed their expression at the mRNA level in our model using qPCR. In contrast, in our model ADAM10^ΔAlb^ mice displayed no significant differences in *Mrp2* liver expression after 4-weeks of CCl_4_-intoxication, as well as in *Mrp3* and *Mrp4* (Fig. S2). Hence, the higher susceptibility of ADAM10^ΔAlb^ towards CCl_4_ treatment is not a consequence of the difference in transporter proteins expression.

### ADAM10^ΔAlb^ biliary epithelial cells are affected after CCl_4_ intoxication

**Figure 6:**
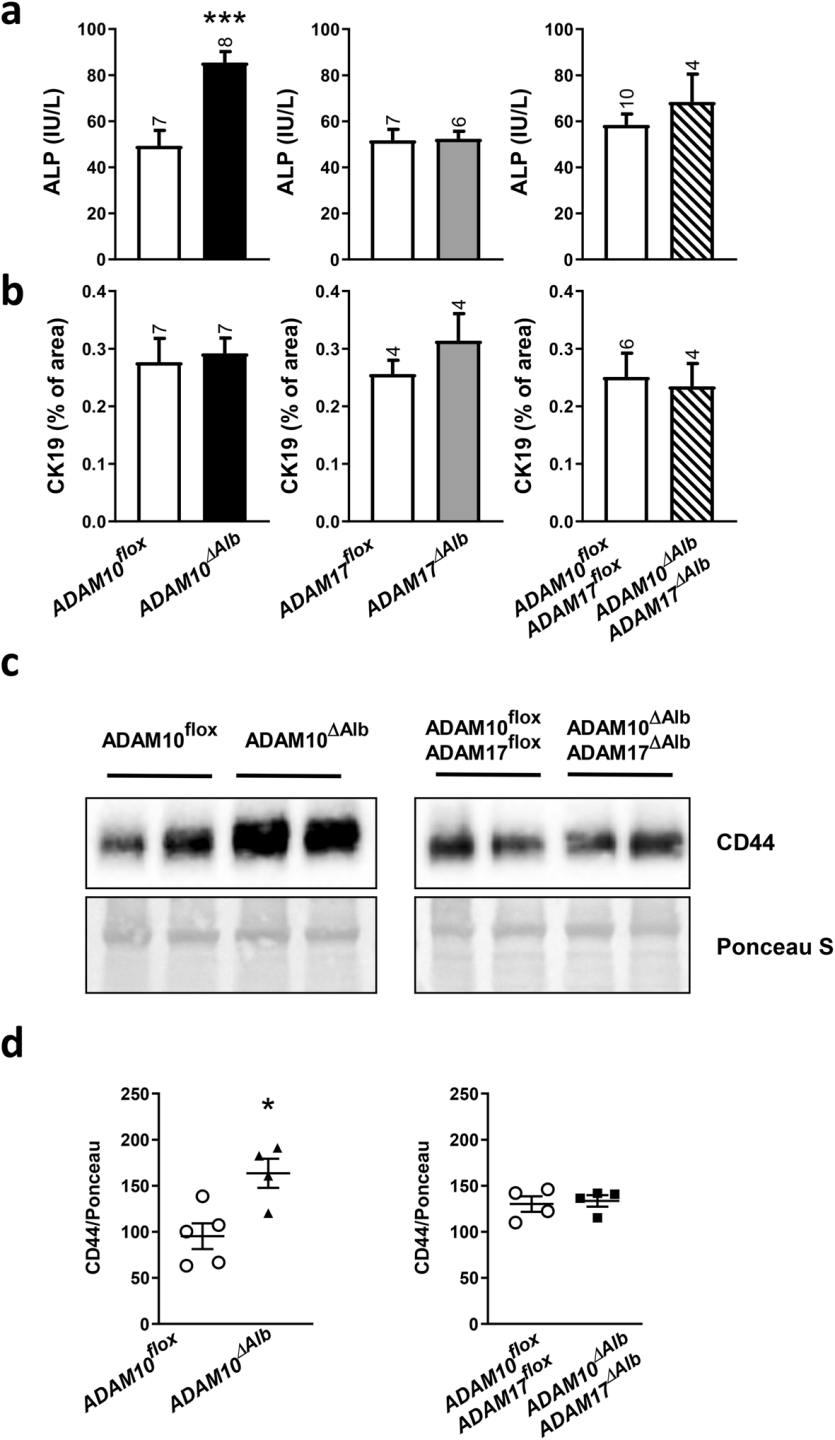
Effect of CCl_4_ intoxication on biliary epithelial cells. (**a**) Serum levels of ALP were elevated in ADAM10^ΔAlb^ mice in comparison to other groups after 4-weeks treatment with CCl_4_. (n indicated above the bars, mean ± SEM, ***p < 0.001). (**b**) Immunohistochemical analysis of CK19 in liver sections did not reveal any differences between studied groups. (n indicated above the bars, mean ± SEM). (**c**) Immunoblotting showed higher levels of CD44 in liver tissue of ADAM10^ΔAlb^ mice after 4-weeks treatment with CCl_4_ (n = 4 mice per group). (**d**) Densitometric evaluation of immunoblots from (c). (mean ± SEM, *p < 0.05).

Serum analysis revealed that ADAM10^ΔAlb^ mice exhibit not only higher levels of hepatocellular damage markers (ALT and AST), but also markers of biliary damage, such as alkaline phosphatase (ALP). ALP activity was elevated in the serum of ADAM10^ΔAlb^ mice after 4 weeks of CCl_4_ treatment in comparison with control littermates, but not in ADAM17^ΔAlb^ or ADAM10^ΔAlb^ADAM17^ΔAlb^ (Fig. 6a). To determine whether elevated ALP levels are connected to hyperproliferation of biliary epithelia, we stained liver sections with CK19, a marker of biliary epithelial cells. ADAM10^ΔAlb^ livers did not differ in the CK19-positive area from controls, and neither did ADAM17^ΔAlb^ or ADAM10^ΔAlb^ADAM17^ΔAlb^ (Fig. 6b). On the other hand, higher levels of CD44 protein, a molecule overexpressed in proliferating biliary epithelium and a substrate of ADAM10, were detected in lysates from ADAM10^ΔAlb^ livers than in controls (Fig. 6c,d). A higher amount of CD44 was not determined in lysates from ADAM10^ΔAlb^ADAM17^ΔAlb^ livers. The observed activation of biliary epithelial cells can be caused by a higher degree of overall liver damage, caused by chronic intoxication in ADAM10^ΔAlb^ mice. Importantly, ADAM17 deficiency overcomes the biliary damage enhanced by ablation of ADAM10.

### Shedding of TNF RI is affected by deficiency of ADAM17 and ADAM10 after CCl_4_ intoxication

**Figure 7:**
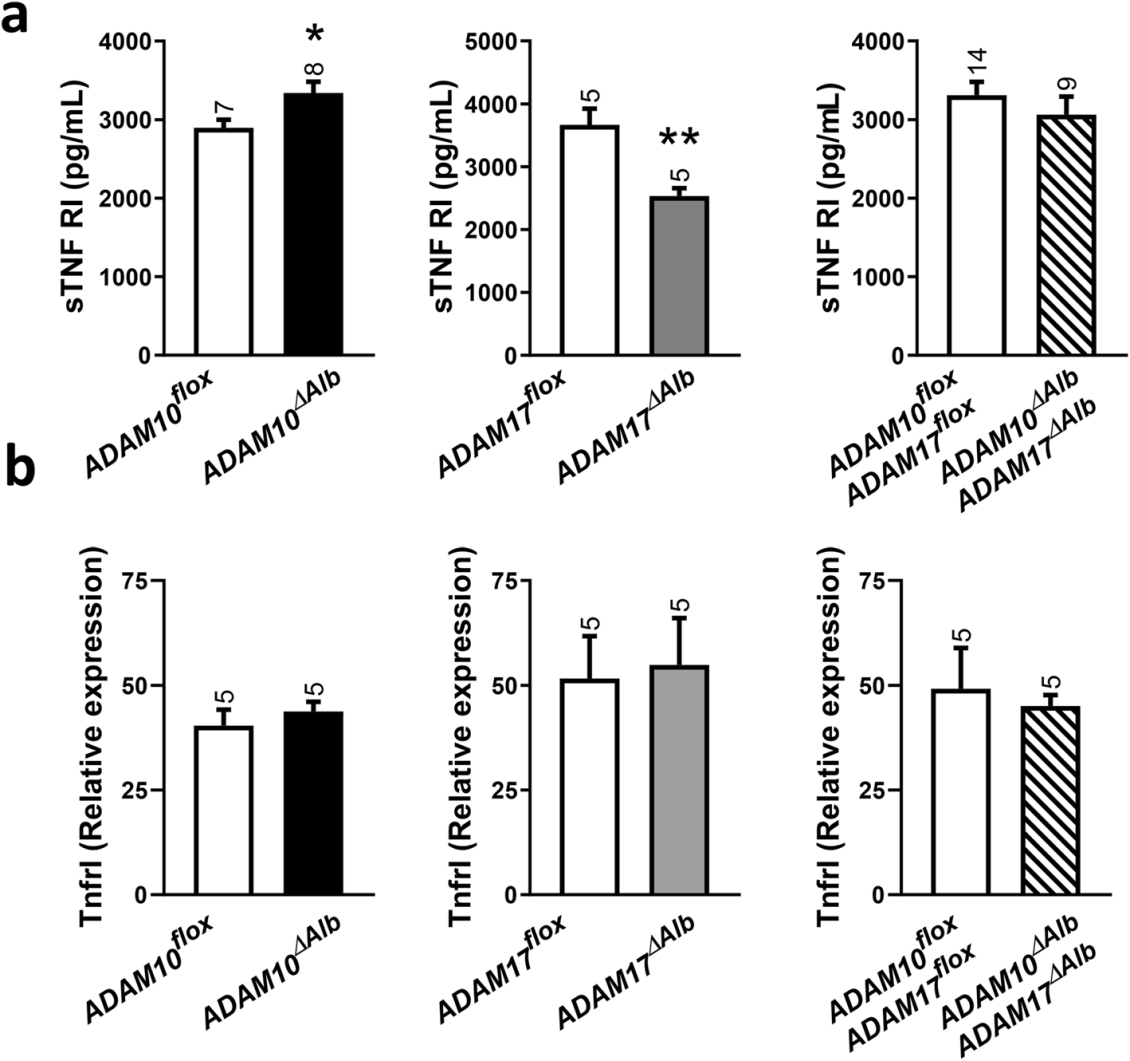
TNF RI shedding in CCl_4_ intoxication. (**a**) Higher shedding of TNF RI into serum was observed 4 weeks after CCl_4_ treatment in all studied groups as determined by ELISA. ADAM17^ΔAlb^ mice had lower levels of soluble TNF RI (sTNF RI) than control littermates, while in ADAM10^ΔAlb^ mice sTNF RI levels were elevated (n indicated above the bars, mean ± SEM, *p < 0.05, **p < 0.01). (**b**) Expression of *TnfrI* transcript in livers after CCL4 treatment was determined by q-PCR. Values were normalized to *Gapdh* and *Hprt.* (n = 5 per group, mean ± SEM).

Previous studies have shown that CCl_4_-induced liver damage is connected with elevated levels of soluble TNF RI (sTNF RI) and TNF RII^35^. In consistency with previous reports, we observed an increase of sTNF RI serum levels after 4 weeks of CCl_4_ treatment in all studied groups in comparison to untreated animals (Fig. 7a). Similarly to the results from partial hepatectomy, ADAM17^ΔAlb^ displayed significantly lower serum levels of sTNF RI than control littermates (Fig. 7a) while ADAM10^ΔAlb^ exhibited the opposite situation with higher levels of TNF RI in serum. Interestingly, ADAM10^ΔAlb^ADAM17^ΔAlb^ mice did not show significant differences to controls. To exclude that identified differences originated from a changed TNF RI expression, we performed qPCR using RNA from whole liver tissue. No differences in *Tnfr1* transcript were found between individual mouse lines (Fig. 7b), indicating that observed differences resulted from alteration in sTNF RI release. A regulatory role of sTNF RI in liver pathological states is emerging^36,37^. Our data suggest that both ADAM10 and ADAM17 influence its release.

## Discussion

ADAM10 and 17 are metalloproteinases that modulate signalling in many physiological processes. For liver regeneration, EGFR and c-Met pathways were identified as the most important ones. Several recent articles showed that only concurrent inactivation of both pathways causes inability of liver to recover from the partial hepatectomy and higher necrosis after CCl_4_ intoxication^7,11,38^. In this study, we demonstrate that both pathways are modulated by ADAM proteases in a pathological liver. Simultaneous hepatocyte-specific deficiency of both ADAM10 and ADAM17 led to lower activation of EGFR as well as to the lower shedding of a c-Met receptor. Thus, initial slower regenerative response after partial hepatectomy, caused by impaired shedding of EGFR activating ligands, was compensated by stronger HGF signalling through c-Met receptor. Furthermore we show, that both proteases are also involved in TNF RI release. ADAM17 directly cleaves the receptor, while ADAM10 deficiency causes elevation of TNF RI serum levels through an unidentified process.

Several EGFR ligands promote the proliferation of hepatocytes. It has been described that expression of HB-EGF, TGF-α^39^, AREG^16^ and epiregulin^40^ is elevated after partial hepatectomy, even though they are not completely fungible^41^. ADAM17 has been described as the main protease in the release of TGF-α, AREG and epiregulin^24^. HB-EGF is shed mainly by ADAM17 and ADAM12, and betacellulin is a substrate of ADAM10^24^. Even though studies in deficient cells indicate that AREG shedding is dependent on ADAM17, not ADAM10 (which we observed as well in shedding from primary hepatocytes), under the specific stimuli ADAM10 can be the major contributor to AREG shedding^28^. In addition, it was already mentioned that ADAM10 dependent shedding of ADAM17 substrates, including TGF-α and HB-EGF, was described in ADAM17-deficient cells upon stimulation^30^. This complicated situation implies that in vivo studies are essential to recognize the contribution of ADAM10 or ADAM17 to EGFR activation in certain physiological/pathophysiological process. We show that the combined deletion of ADAM10 and ADAM17 in hepatocytes leads to lower levels of EGFR activating factors in the serum from peripheral blood after 2/3 partial hepatectomy. This was in line with lower levels of phosphorylated AKT in livers of ADAM10^ΔAlb^ADAM17^ΔAlb^ mice. None of these differences was observed in single deficient mice. Altogether this indicates that hepatocytes alone are a significant source of growth factors that modulate their cell cycle. Similar results were described by McMahan et al.^29^ in hepatocyte-specific ADAM17-deficient mice, in which dramatically reduced levels of AREG were observed. However, AREG levels in their study were measured in liver tissue lysates and therefore represented the expression of a protein, not its shed variant. In our model we did not observe differences of expression in AREG protein but we clearly showed that the deletion of ADAM proteases influenced release of EGFR ligands to the serum. Our data indicate that not only ADAM17, but also ADAM10 is a regulator of EGFR ligand shedding after partial hepatectomy. Of note is that factors released from hepatocytes can potentially influence also other liver cell types, e.g. hepatic stellate cells. This may have a negative impact in conditions such as liver fibrosis when enhanced hepatic stellate cells proliferation is not desired.

Similarly to EGFR, HGF signalling through c-Met receptor is another independent pathway leading to the proliferation of hepatocytes^7,42^. In our model, serum HGF was strongly increased after partial hepatectomy. On the other hand, we showed that shedding of c-Met was also elevated, especially in the first hours after the hepatectomy, which is probably part of a regulatory mechanism of HGF signalling. We observed that isolated primary ADAM10 and ADAM17 double-deficient hepatocytes had a more prominent response to HGF treatment. We concluded that these differences in HGF response were caused by higher availability of c-Met receptor on cell surface due to its limited shedding in the combined absence of ADAM10 and ADAM17. In summary, simultaneous deletion of ADAM10 and ADAM17 driven by the albumin promoter leads to hampered release of EGFR ligands, but enhanced HGF/c-Met signalling in regenerating hepatocytes. As a result, ADAM10^ΔAlb^ADAM17^ΔAlb^ mice did not show any alteration from control littermates 40 h post hepatectomy, hepatocytes proceeded towards proliferation and liver weights increased normally. Shedding of EGFR ligands and c-Met was not significantly inhibited in the absence of ADAM10 or ADAM17 individually.

We have reported previously that liver-specific ADAM10-deficient mice, with stronger expression of Cre (Alb-Cre homozygotes or α-fetoprotein-Cre hemizygotes), developed spontaneous fibrosis^22^. This was not the case in mice used in this study (Alb-Cre hemizygotes). ADAM10^ΔAlb^ mice were monitored up to 33 weeks of age, and none of the fibrotic markers were increased in unchallenged animals. The lower expression levels of Cre in hemizygote animals resulted in sustained expression of ADAM10 in a negligible proportion of hepatocytes, which was sufficient to protect from development of spontaneous fibrosis. However, ADAM10^ΔAlb^ mice were much more susceptible to liver damage than control littermates when exposed to the toxic agent. Difference was not apparent after acute intoxication (single dose of CCl_4_) but was clearly distinct after 4 weeks of chronic CCl_4_ treatment. The reason for the higher susceptibility of ADAM10-deficient mice towards CCl_4_ intoxication is still not clear. It was shown that ADAM10 directly influences the expression of bile acid transporters, e.g. MRP2^22^, which play an important role in removal of toxic products generated in hepatocytes after CCl_4_ treatment. However, we did not find the difference in *Mrp2* mRNA expression*,* nor in the expression of *Mrp3* and *Mrp4* which are involved in CCl_4_ detoxication^43^. The difference in *Mrp2* expression between two studies may have arisen due to the differing nature of the injuries evoked in the studies, as we used toxin-induced injury, while model of Muller et al.^22^ resulted from an inner imbalance of liver homeostasis.

Not only levels of ALT and AST were increased in ADAM10^ΔAlb^, but also ALP. ALP in liver damage is connected with activated biliary epithelia. This is in line with previously published data which suggest that ADAM10-deficient mice have increased ductular reaction^22^. Consistently with this, we found that ADAM10^ΔAlb^ had increased protein levels of CD44 in liver lysates. Biliary epithelial cell are a prominent source of CD44 in liver^44^, and its expression is connected to the proliferation of biliary epithelia. The identified increase of CD44 levels could be directly linked to ADAM10 deficiency, as ADAM10 is the primary sheddase for CD44^44^.

Interestingly, the hepatocyte damage in ADAM10^ΔAlb^ADAM17^ΔAlb^ and ADAM17^ΔAlb^ mice was comparable to control littermates. Thus, ADAM17 elimination was not protective in CCl_4_ induced liver fibrosis when functional ADAM10 was present, but alleviated aggravating impact of ADAM10 deficiency on fibrosis development. We have shown previously^32^ that ADAM17 inhibition by ursodeoxycholic acid is protective in model of cholestatic injury. This effect is similar to what we see in ADAM10^ΔAlb^ADAM17^ΔAlb^_,_ double deficient mice do not have increased levels of ALP in serum nor do they have higher levels of CD44 in liver. Thus, these results further support our finding of protective effect of ADAM17 inhibition in cholestatic injury. In contrast, recent work from Sundaram et al.^45^, showed that impaired ADAM17 maturation exacerbated bile duct obstruction induced fibrosis. The main difference between their work and our present findings is the cell type affected by genetic models. Our model leads to specific ablation of ADAM17 in hepatocytes and biliary epithelial cells. Sundaram et al. studied mice with whole body deficiency of iRhom2 that leads to the inhibition of ADAM17 in cell types, in which iRhom2 governs maturation of ADAM17. They explained their phenotype through the suppression of ADAM17 in hepatic stellate cells, a cell type which was not affected in our genetic model. This shows that the modulation of ADAM17 activity on different cell types within the liver can lead to opposite pathological consequences.

Role of ADAM17 in TNF-α shedding is very well-known. However, growing number of evidence suggest the importance of TNF receptors shedding in the regulation of TNF-α signalling^35^. In our study, we found that ADAM17 deficiency in hepatocytes leads to lower levels of TNFRI in serum in both studied liver challenges, as expected. Surprisingly, ADAM10-deficient animals consistently show higher levels of TNF RI in serum than their control littermates. Same results were confirmed in vitro on primary hepatocytes, suggesting elevated serum levels of sTNF RI in vivo had a source in ADAM10-deficient hepatocytes. This effect is most likely not caused by compensational activation of ADAM17, as the other substrate of ADAM17, AREG, was not elevated in the medium of ADAM10-deficient cells. Moreover, in an unchallenged state, also ADAM10^ΔAlb^ADAM17^ΔAlb^ mice exhibit increased shedding of TNF RI, thus this effect is observed even when ADAM17 was missing. During the liver challenge, double deficient mice were comparable to ADAM17^ΔAlb^ or control littermates depending on the specific time point. This nicely shows that the activity of ADAM17 is induced in liver pathological states and the lack of this induction becomes evident in ADAM10^ΔAlb^ADAM17^ΔAlb^ after the challenge, while in unchallenged state effect of ADAM10-deficiencyemerges in respect to TNF RI shedding. Besides proteolytic shedding, soluble TNF RI can be released into serum by exosome-like vesicles^46^. It would be worth to investigate whether ADAM10 deficiency alters this export.

TNF RI was described as a major negative regulator of hepatocyte repopulation after toxin-induced injury^34^. As ADAM10^ΔAlb^ sheds more of a TNF RI, it can be expected that TNF-α signalling through TNF RI is hampered. As a result, ADAM10-deficiency hepatocytes could overcome the inhibitory effect of TNF RI on hepatocyte proliferation. Interestingly, we showed that ADAM10^ΔAlb^ after CCl_4_ intoxication exhibited increased hepatocyte proliferation. There was also a tendency in increased AKT activation 6 h after hepatectomy, though proliferation in later time point was not changed. Moreover, in the previously published model of ADAM10-deficient mice with spontaneous fibrosis development^22^, increased proliferation of hepatocytes was indicated as a part of a mechanism of homeostasis imbalance and fibrosis development. TNF RI signalling could be another part fulfilling the picture of how ADAM10 regulates the homeostasis of the liver. In addition, as ADAM10 and ADAM17 counteract in TNF RI release, this could be part of the protective mechanisms which was identified in ADAM10^ΔAlb^ADAM17^ΔAlb^ in comparison to ADAM10^ΔAlb^ after induced fibrosis.

Altogether our data show, that hepatocyte-derived ADAM10 and ADAM17 contribute to the regulation of several distinct pathways, i.e. EGFR, HGF and TNF RI, during liver injury and regeneration. Potential therapies based on inhibition of ADAM10 and ADAM17 should carefully consider various roles of these proteases in different cell types.

## Materials and methods

### Mouse models

Mouse lines ADAM10 flox on 129/C57Bl/6 background^47^ (kindly provided by Dr. Paul Saftig, CAU Kiel) and ADAM17 flox C57Bl/6J^48^ (kindly provided by Dr. Carl Blobel, HSS Research institute) were first backcrossed to C57Bl/6 N. N4 generations were crossed together to create ADAM10 flox ADAM17 flox line. All three floxed stains were crossed to line expressing Cre under the control of albumin promoter, Alb-Cre (B6.Cg-Tg(Alb-cre)21Mgn/J; The Jackson Laboratory). For liver challenges 9–14 weeks old males were used. Animals used in all experiments were homozygous for flox alleles and hemizygous for Cre recombinase and were termed ADAM10^ΔAlb^, ADAM17^ΔAlb^, ADAM10^ΔAlb^ ADAM17^ΔAlb^. Littermates without Cre gene were used as a control group in each line, marked as ADAM10^flox^, ADAM17^flox^, ADAM10^flox^ ADAM17^flox^.

Model of 2/3 partial hepatectomy was performed as described previously^49^ with slight modifications. Briefly, mice were anesthetized with mixture of Zoletil (Virbac, Carros, France) and Rometar (Bioveta, Ivanovice, Czech Republic), frontal lobes and left lateral lobe are ligated with silk suture and subsequently removed. For sample harvesting mice were anesthetized at indicated time points after the surgery, blood was collected from orbital plexus, animals were subsequently euthanized by cervical dislocation and liver samples were taken from defined areas of remaining lobes – one piece was fixed for histological analysis (see below) and several smaller pieces were snap frozen and kept in -80 °C for RNA and protein isolation.

To induce fibrogenesis, mice were injected intraperitoneally with 1 µl/g of CCl_4_ (Sigma-Aldrich, Darmstadt, Germany) diluted 1:3 with olive oil twice a week for 4 weeks. Liver and blood samples were collected 48 h after the last injection as described for partial hepatectomy model.

The animals were handled in accordance with the Guide for the Care and Use of Laboratory Animals, approved by the Animal Care and Use Committee of the Academy of Sciences of the Czech Republic. Mice were kept under standard laboratory conditions with free access to food and water.

### Primary hepatocyte isolation and treatment

Hepatocytes were isolated by perfusion with collagenase I as described^50^. Hepatocytes were plated on collagen I (0.3 mg/mL) and let to attach in DMEM containing 10% fetal bovine serum (FBS), 1% penicillin–streptomycin and 0.08 U/mL insulin (culture medium) for 24 h before proceeding with experiments.

For shedding assay, cells were maintained in fresh culture medium for 6 h. Harvested medium was cleared from debris by centrifugation (10,000 × g/10 min) and analysed by ELISA. For HGF stimulation, cells were kept in culture media without FBS for 6 h, then incubated with recombinant human HGF (20 ng/mL; R&D Systems) in fresh medium for 30 min. After stimulation cells were immediately lysed with RIPA buffer (25 mM Tris, 150 mM NaCl, 10 mM EDTA, 1% triton X-100, 1% DOC, 0.1% SDS, Complete® inhibitors (Roche), 1 mM ortho-vanadate, 2.5 mM sodium pyrophosphate, 1 mM β-glycerolphosphate).

For stimulation of primary hepatocytes with mouse serum, cells were kept in culture media without FBS for 6 h, then treated for 30 min with DMEM medium supplemented with 10% of mouse sera and subsequently lysed with RIPA buffer. Samples were further processed for immunoblotting and ELISA.

### Histological analysis

Specimens were fixed in 4% buffered formaldehyde, embedded in paraffin, sectioned, and stained with hematoxylin and eosin (H&E), Sirius Red^51^, or processed for immunohistochemistry. To visualize proliferating cells, sections were stained with antibodies against Ki-67 (Dako, Santa Clara, CA) and PCNA (Santa Cruz Biotechnology, Dallas, TX). For the analysis, 10 optical fields (original magnification 400 x) were taken per animal using Axio Imager Z2 (Zeiss, Oberkochen, Germany) with Zen 3.1 software (https://www.zeiss.com/microscopy/int/products/microscope-software/zen.html) and positively stained nuclei were counted. Sirius red staining (original magnification 200 x) was quantified using Fiji/ImageJ software^52^.

### Analysis of serum parameters

Collected blood was allowed to cloth for 1 h in 37 °C, spun down (10,000 g /20 min), and cleared serum was kept frozen before further analysis. Serum levels of IL-6, KC, soluble c-Met and soluble TNF- receptor I were assayed by ELISA using antibodies from R&D Systems (Minneapolis, MN). Activities of alanine aminotransferase (ALT), aspartate aminotransferase (AST) and alkaline phosphatase (ALP) were assessed using kits from Roche Diagnostics (Basel, Switzerland).

### Immunoblotting

Frozen pieces of liver tissues were homogenized in RIPA buffer using TissueLyzer II (Qiagen, Hilden, Germany). Equal amounts of protein as assessed by BCA assay were resolved on SDS polyacrylamide gels and transferred to nitrocellulose membranes; proteins were detected using antibodies against phospho c-Met, phospho-AKT, AKT, phospho-EGFR, EGFR (all Cell Signaling Technology, Danvers, MA) and GAPDH (Sigma-Aldrich, Darmstadt, Germany) as loading control. Visualisation was done using LAS-3000 (Fujifilm, Tokyo, Japan) except for immunoblots from Fig. 6c, which were imaged by ChemiDoc MP Imaging System with Image Lab software 6.0.1. (Bio-Rad Laboratories, Hercules, CA, https://www.bio-rad.com/en-cz/product/image-lab-software?ID=KRE6P5E8Z). Densitometric analysis was performed by AIDA Image Analyzer software version 3.1 (Raytest, Straubenhardt, Germany, www.elysia-raytest.com/en/cataloglight/c30~aida-image-analysis-software).

### Quantitative reverse-transcriptase polymerase chain reaction (qRT-PCR)

Total RNA was isolated from piece of snap frozen mouse liver tissue by TRI Reagent® (Sigma-Aldrich). mRNA was transcribed using polyT primers and MLV-RT (Promega, Fitchburg, WI). For RT reaction, SYBR® Green JumpStart™ Taq ReadyMix™ (Sigma-Aldrich) was used. Primers for amplification of mRNA were used as follows: *Adam10* (5′-GCTGGGAGGTCAGTATGGAA-3′, 5′-TGGTCCTCATGTGAGACTGC-3′), *Adam17* (5′-CCACCACCACGACTCTCAAG-3′, 5′-CAGTCTGCGACACACTTAGAAAC-3′), *TnfrI* (5′-TAACTGCCATGCAGGGTTCT-3′, 5′-CTGGGGGTTTGTGACATTTG-3′). Data were normalized to *Gapdh* (5′-CGTCCCGTAGACAAAATGGT-3′, 5′-TTGATGGCAACAATCTCCAC-3′) and *Hprt* (5′-TCCTCCTCAGACCGCTTTT-3′, 5′-CCTGGTTCATCATCGCTAAT-3′).

### Statistical analysis

Statistical analysis was performed in GraphPad Prism version 8.1.1 (GraphPad Software, San Diego, California USA, www.graphpad.com). Normal distribution was evaluated by Shapiro–Wilk test. Normally distributed data were analysed using the Student´s two-tailed t-test. Data without normal distribution were analysed by the Mann–Whitney U test. In Fig. 4e multiple comparisons were performed using two-way ANOVA with Sidak’s correction (comparison of the differences between deficient mice and their control littermates in individual time point) or Dunnett’s post hoc test (comparison of increase in one group of animals in the different time points). Figures 2d and 4e were analysed using one-way ANOVA with Dunnett’s post hoc test. *p* values were considered significant under 0.05: *p < 0.05, **p < 0.01, ***p < 0.001. Data are shown as mean ± SEM, unless stated otherwise. Numbers of used mice from each line per experiment are indicated in each figure. Data from primary hepatocytes were collected from three independent isolations.

### Compliance statement

The study was carried out in compliance with the ARRIVE guidelines.

## Supplementary Information

Supplementary Information.
