# Supplementary material for: ADAM10 and ADAM17 regulate EGFR, c-Met and TNF RI signalling in liver regeneration and fibrosis
Source: Sci Rep. 2021 Jun 1;11:11414. doi: 10.1038/s41598-021-90716-3 (PMC8169909; doi:10.1038/s41598-021-90716-3)
Supplement: Supplementary file 1 — Supplementary Information. [file 41598_2021_90716_MOESM1_ESM.pdf]

ADAM10 and ADAM17 in liver regeneration and fibrosis: regulation of EGFR, c-Met and TNF RI signalling

Olga Zbodakova[1*], Karel Chalupsky[1,2], Lenka Sarnova[1,3], Petr Kasparek[1,2], Marketa Jirouskova[1,3], Martin Gregor[1,3] and Radislav Sedlacek[1,2 *]

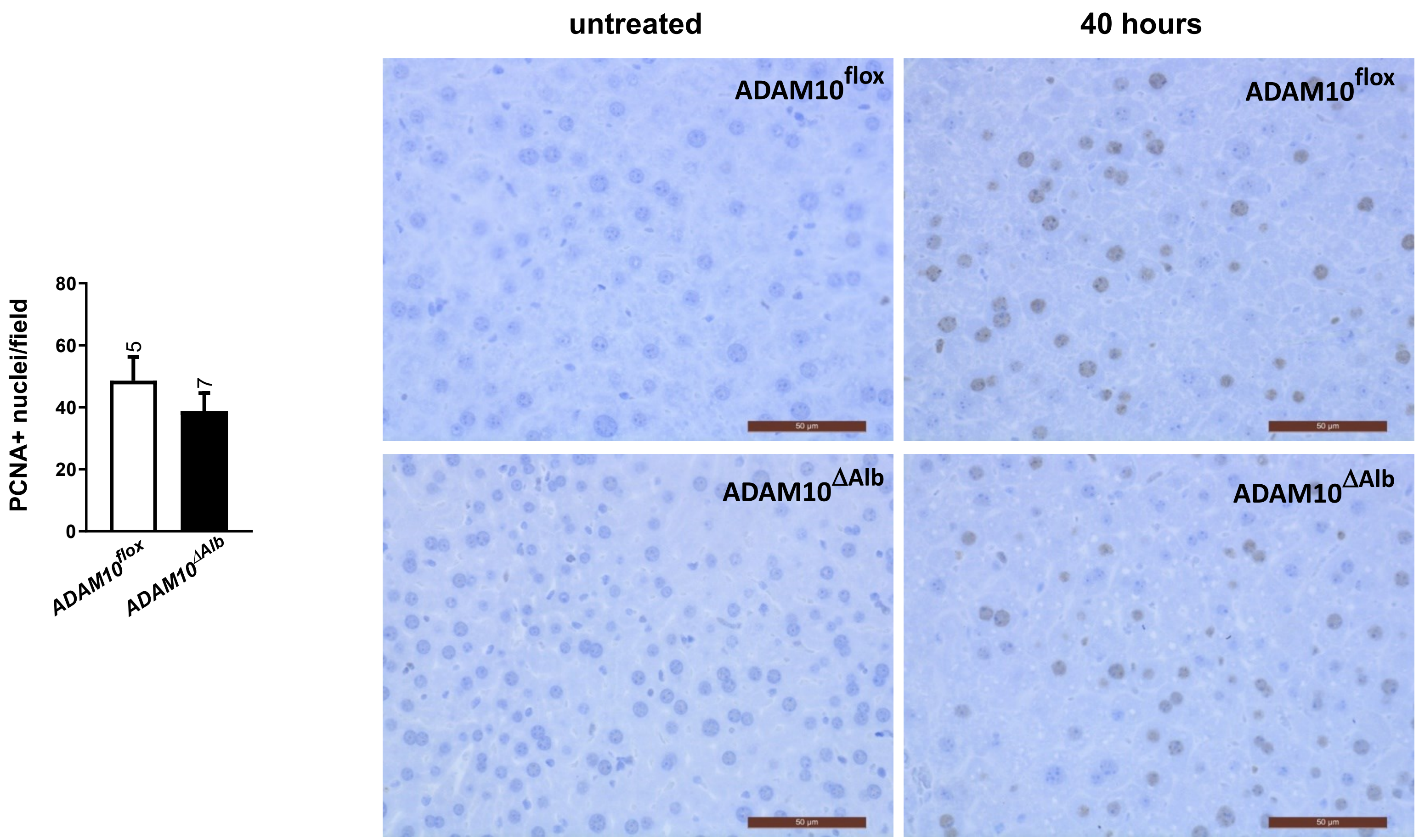

Fig. S1. Analysis of hepatocyte proliferation in ADAM10$^{\Delta Alb}$ 40 hours post hepatectomy. Immunohistological staining of PCNA revealed no differences in hepatocyte proliferation between ADAM10$^{\Delta Alb}$ and their control littermates. (n indicated above the bar, mean ± SEM)

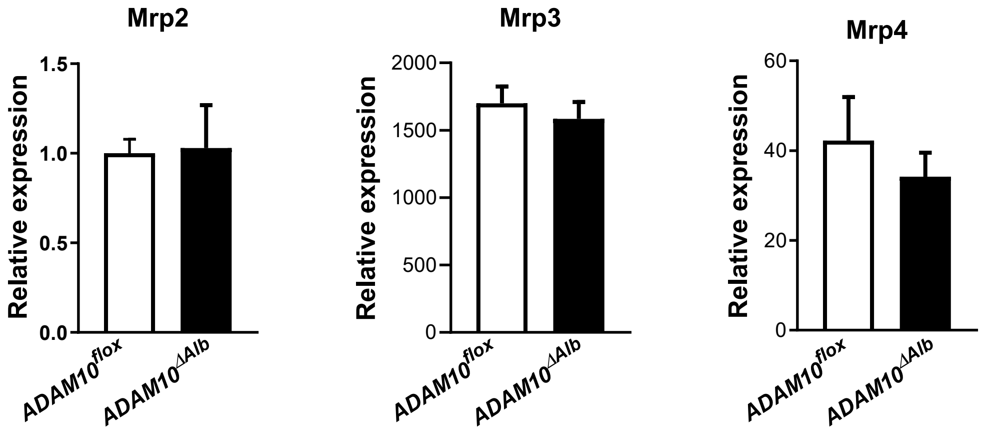

Fig. S2. mRNA expression of bile acid transporters in liver tissue after CCl_4 intoxication. No differences in mRNA levels of *Mrp2*, *Mrp3* and *Mrp4* between lines were detected. Data were normalized to *Gapdh* and *Hprt* (n=5 mice per group, mean ± SEM).

Original pictures Fig 1.

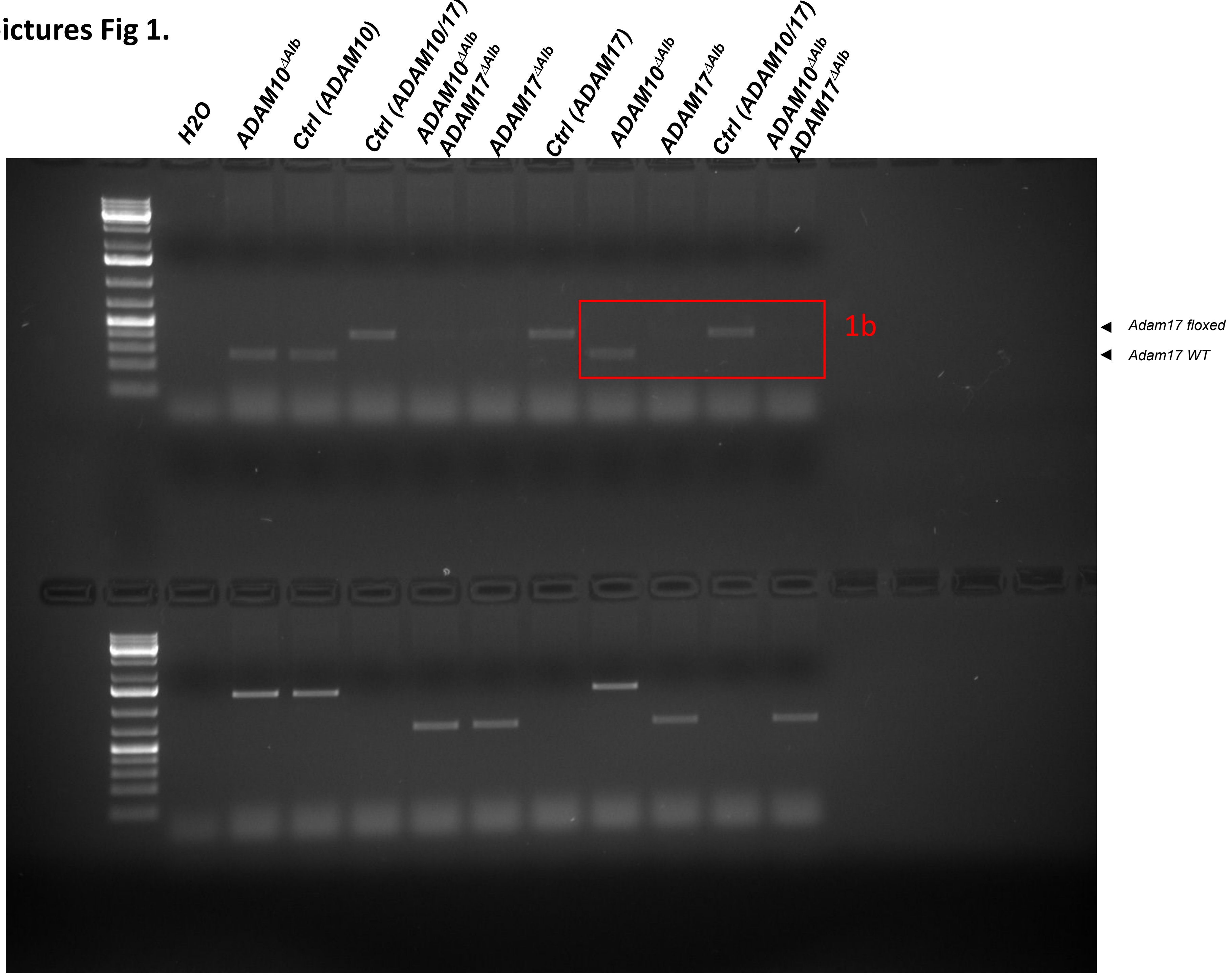

Original pictures Fig 1.

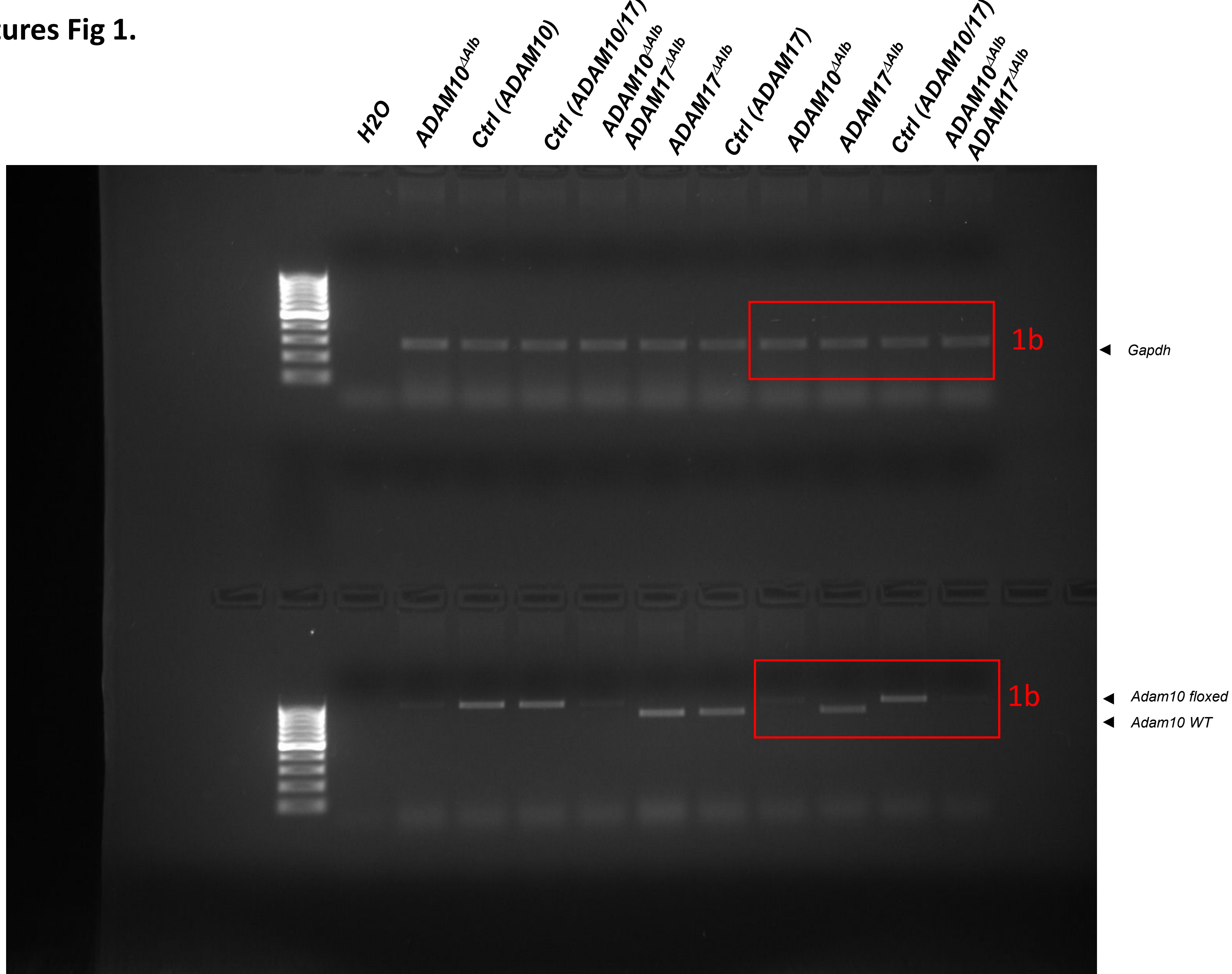

Original pictures Fig 2.

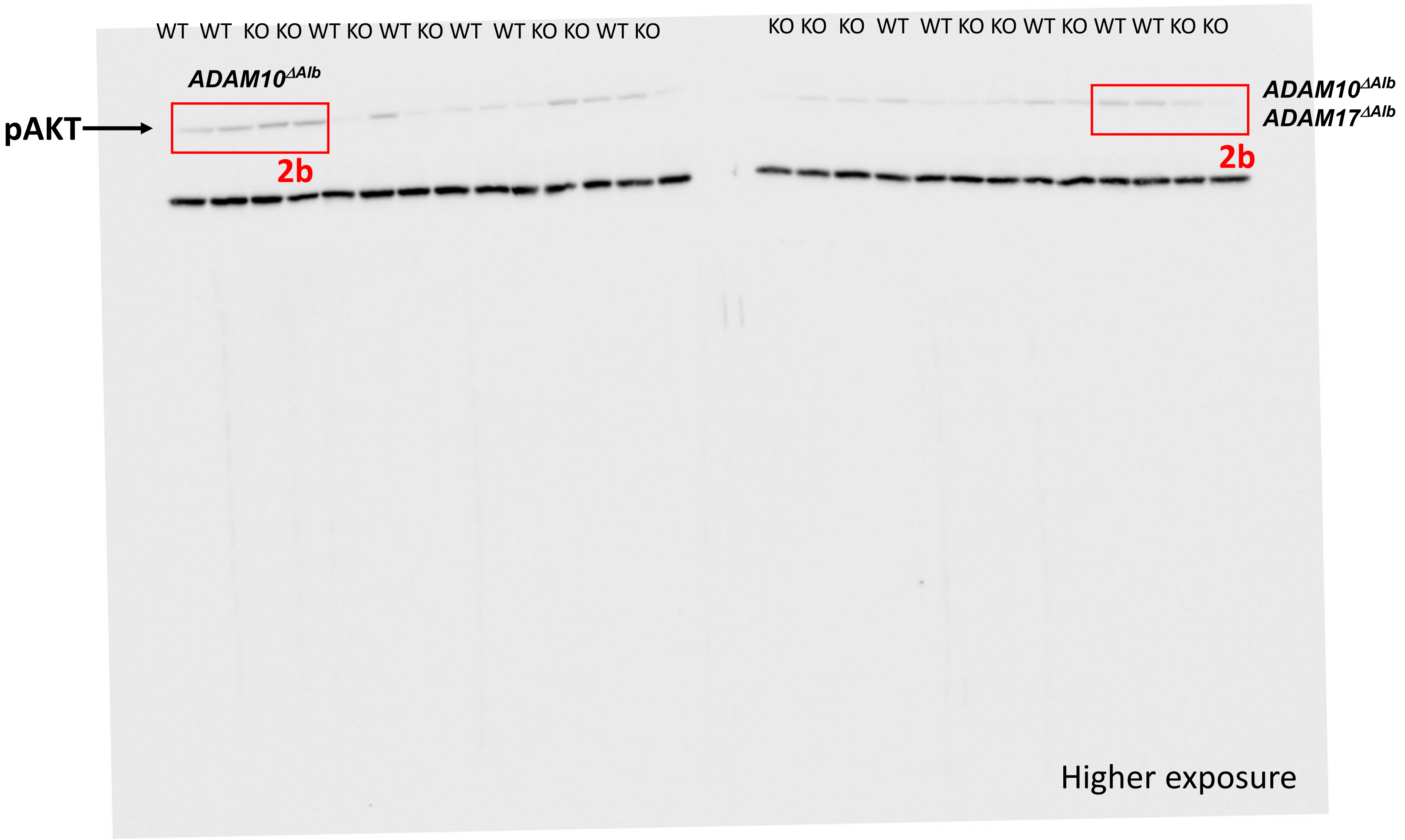

Original pictures Fig 2.

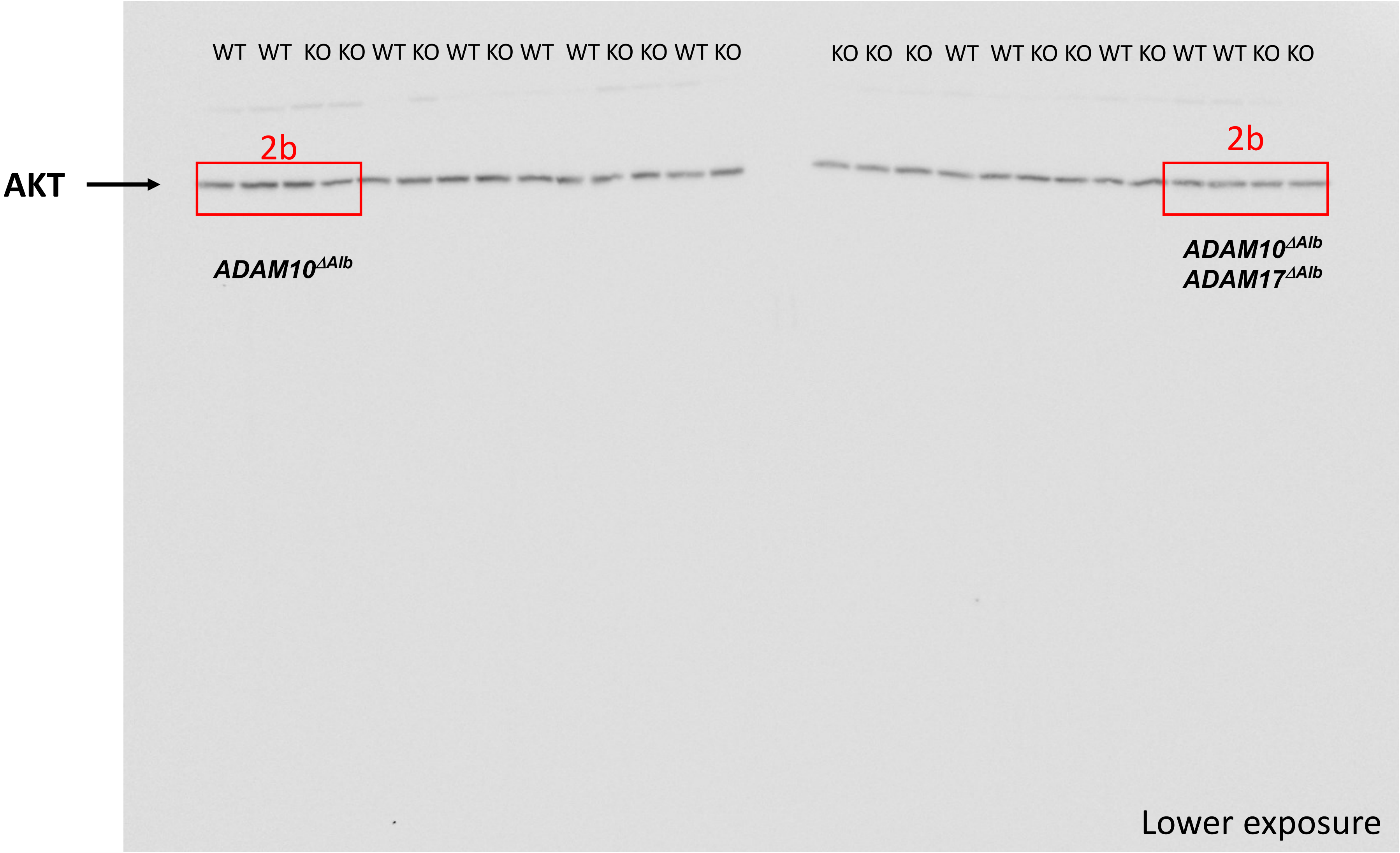

Original pictures Fig 2.

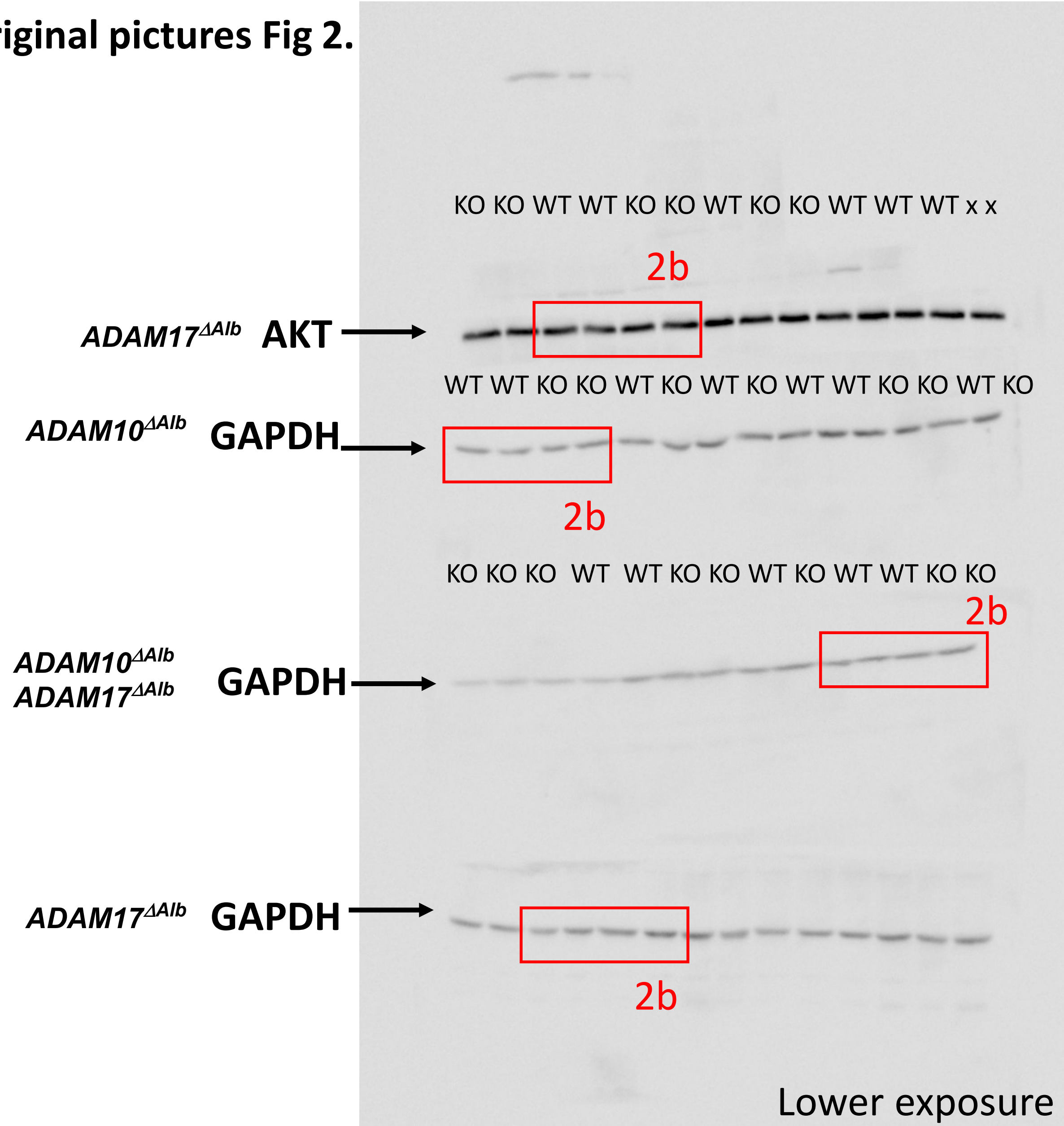

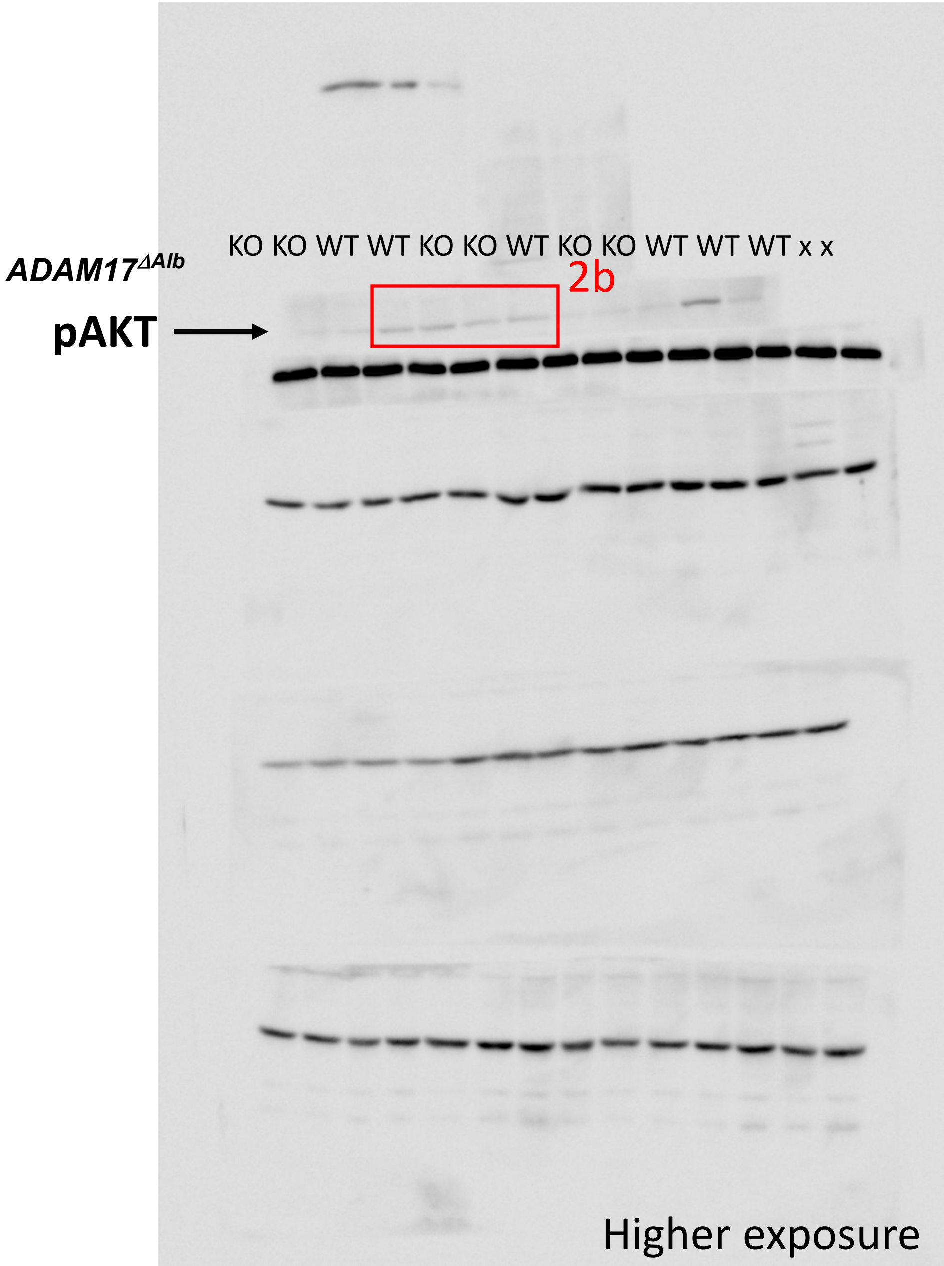

Original pictures Fig 2.

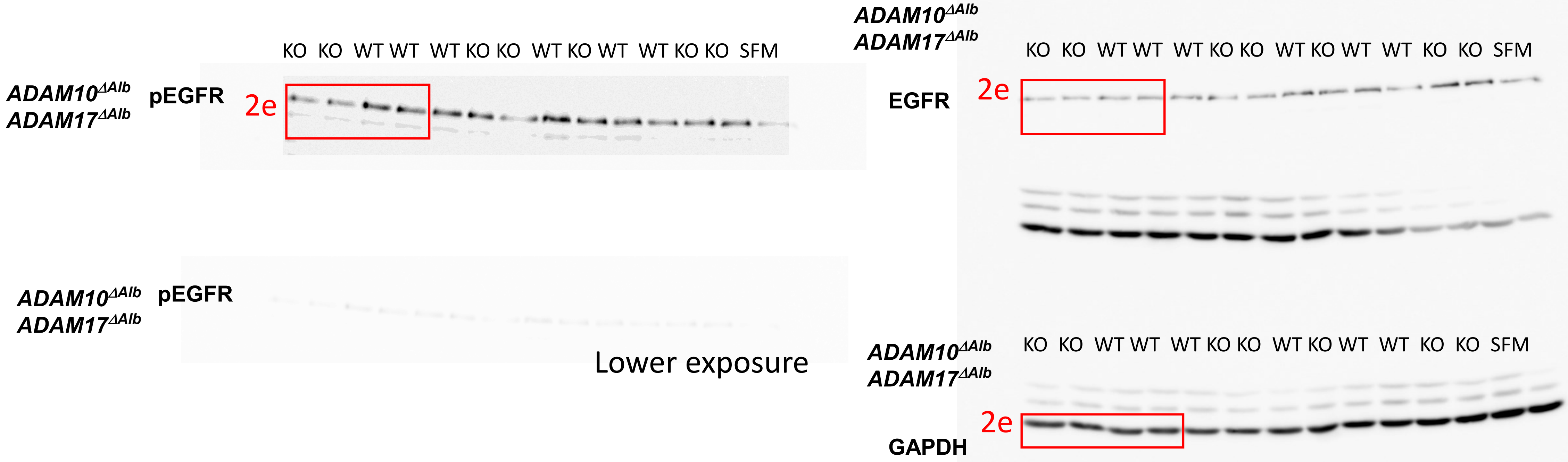

Original pictures Fig 2.

pEGFR

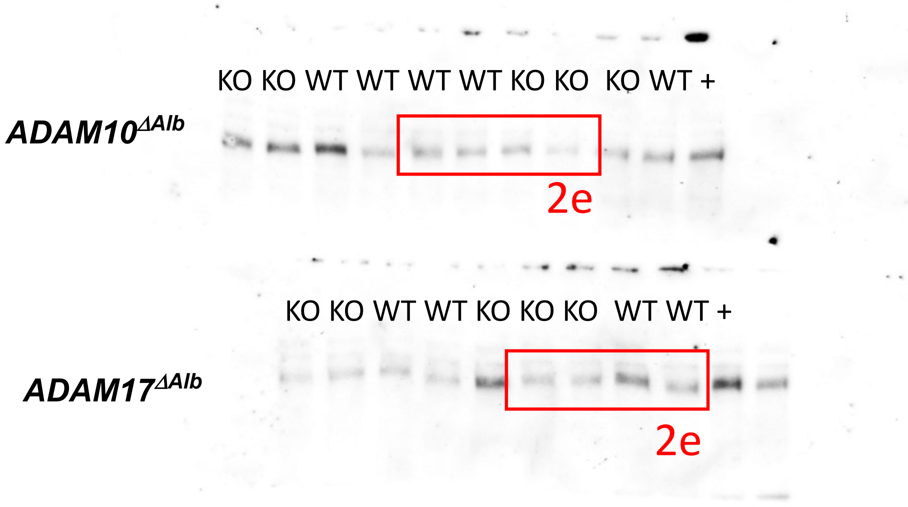

GAPDH

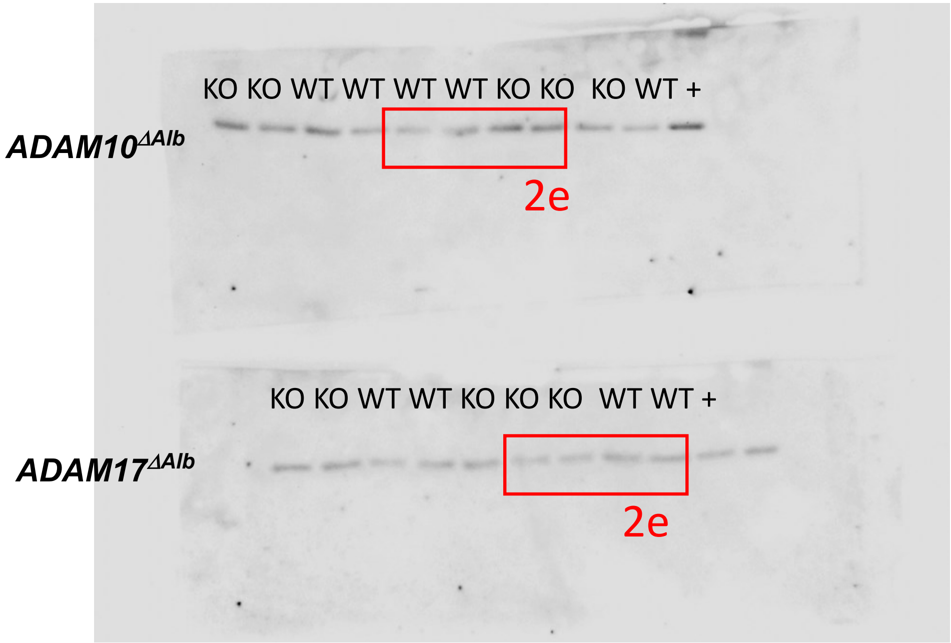

GAPDH

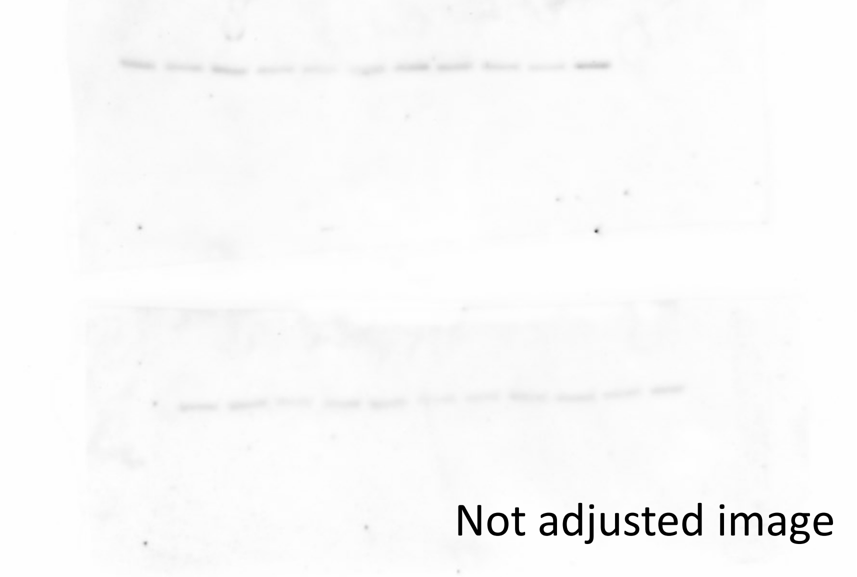

Not adjusted image

Original pictures Fig 4.

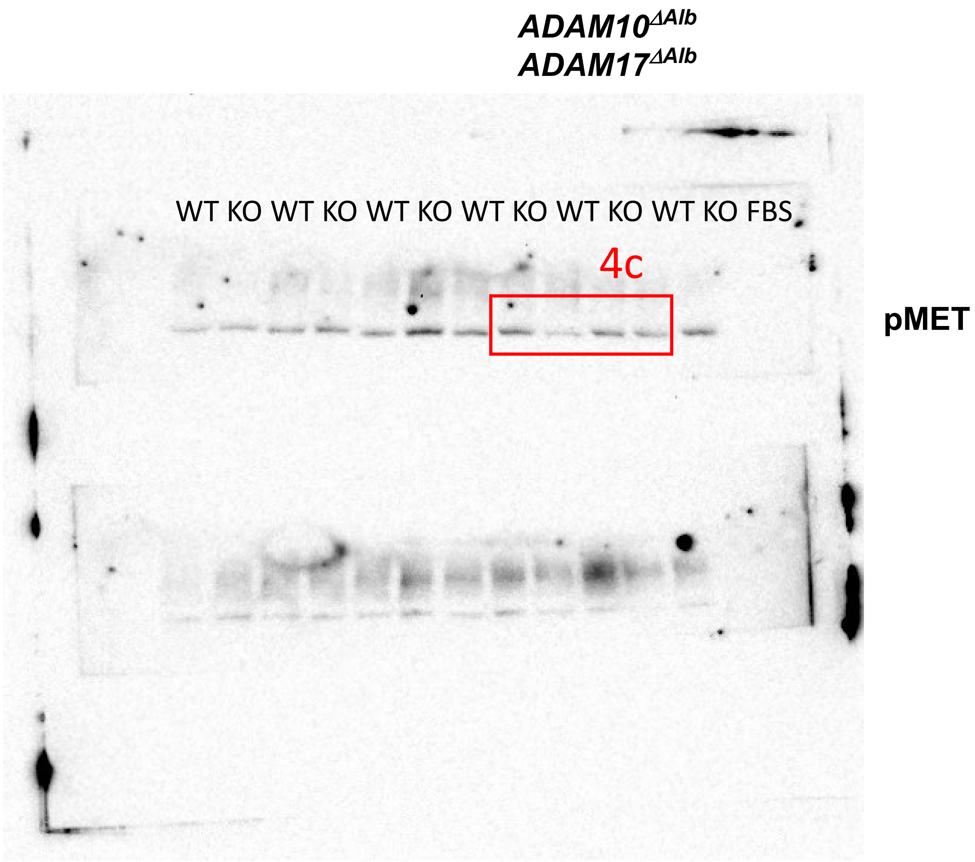

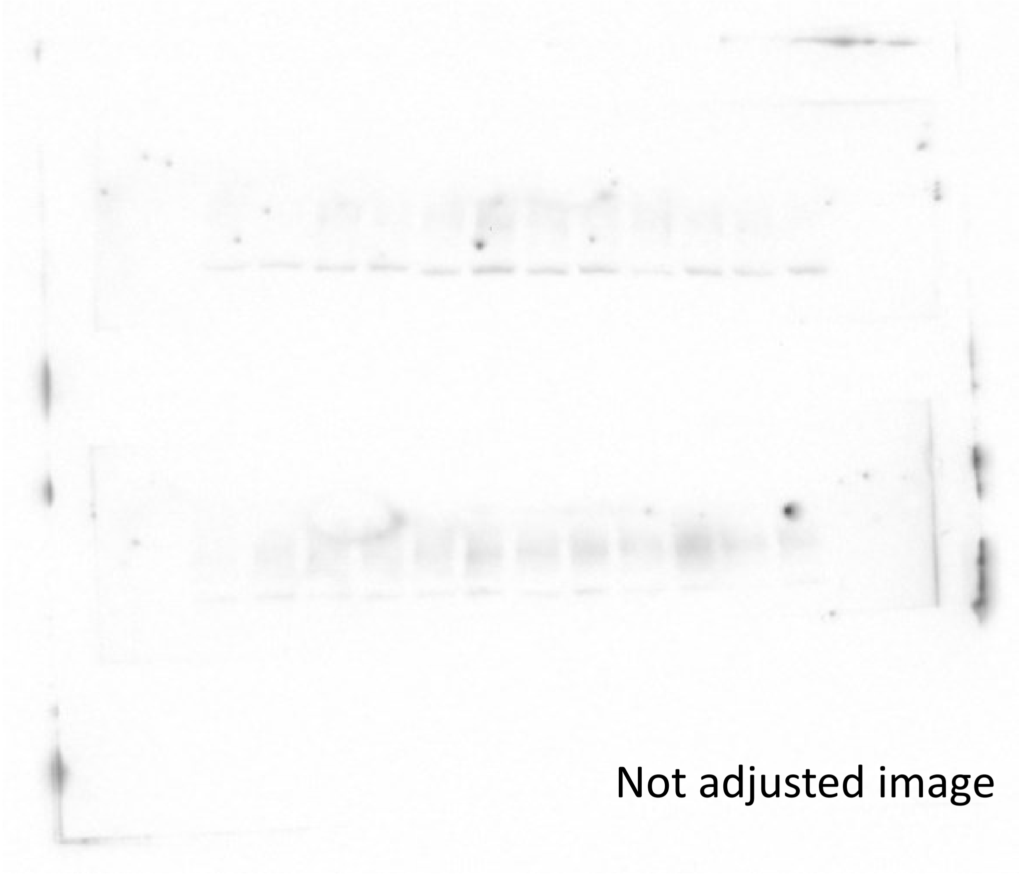

Not adjusted image

Original pictures Fig 4.

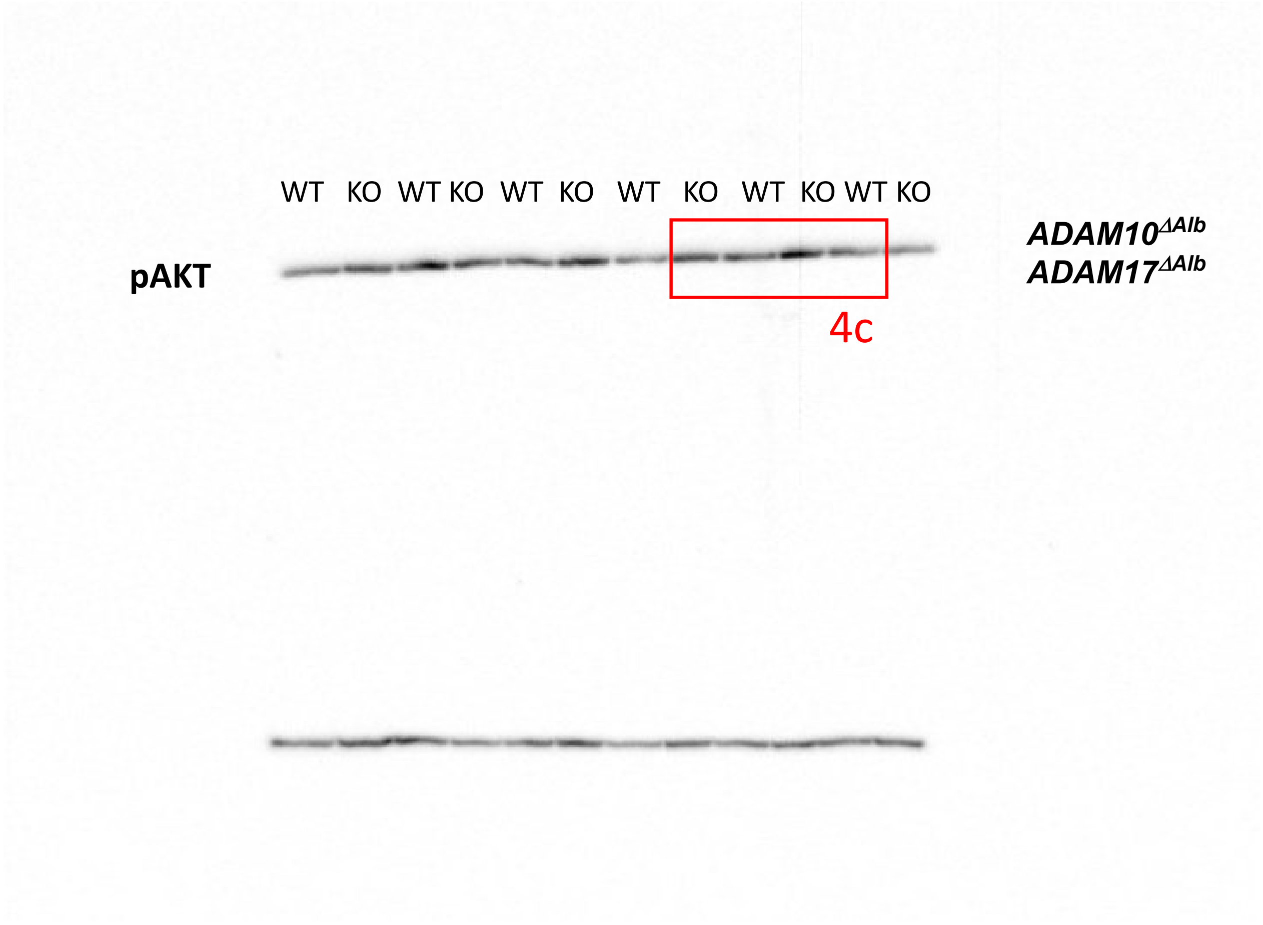

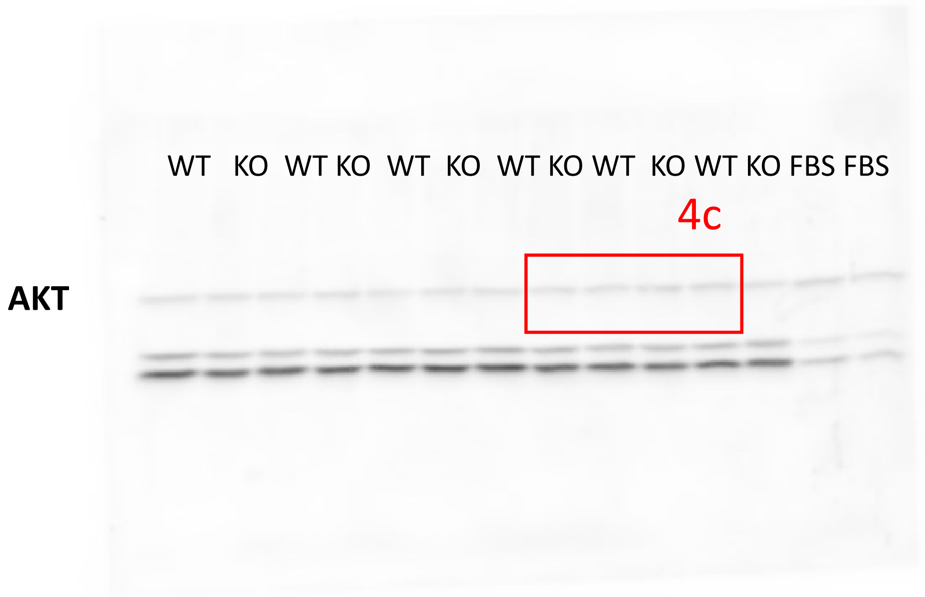

Original pictures Fig 4.

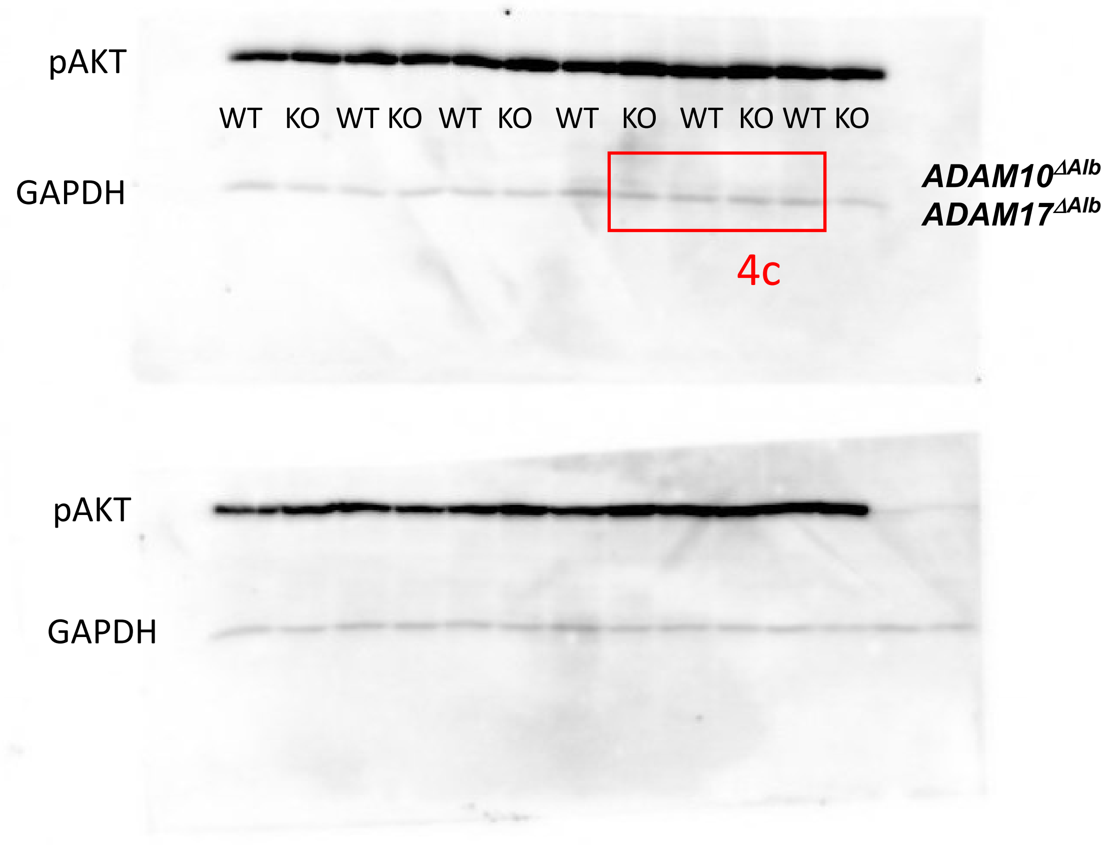

Reprobed membrane

Original pictures Fig 6.

CD44

Ponceau S

ADAM10

ADAM10$^{\Delta Alb}$ KO KO WT WT KO KO KO WT WT WT

6c

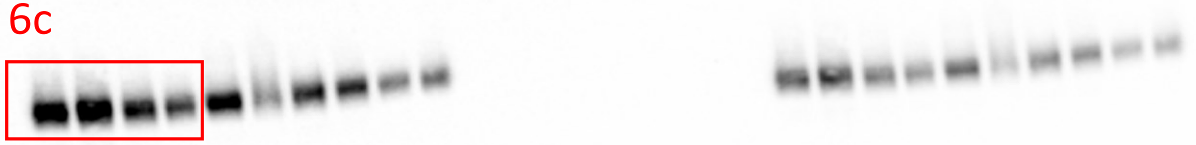

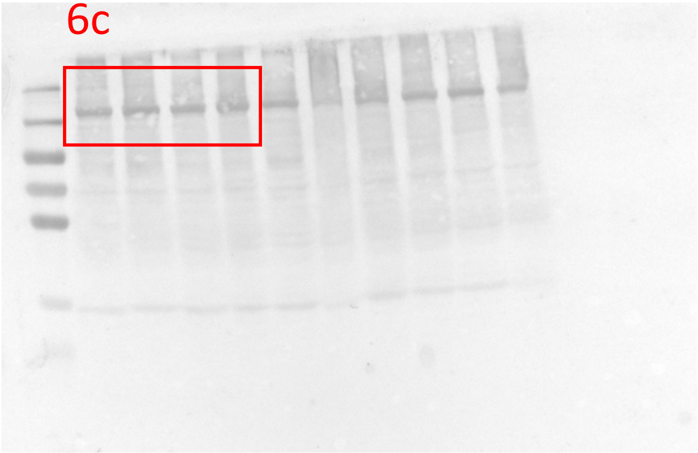

ADAM10$^{\Delta Alb}$
ADAM17$^{\Delta Alb}$ KO KO WT WT KO KO WT WT

6c

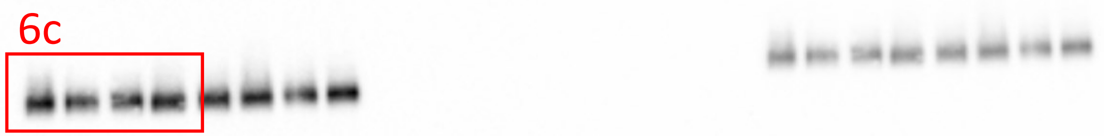

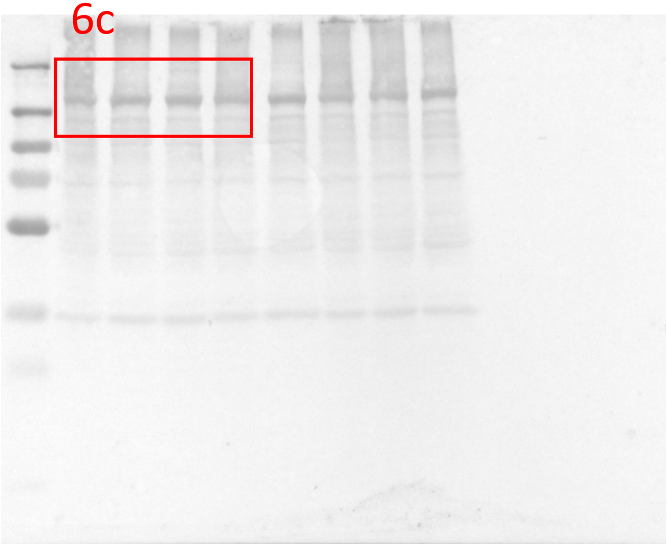

Not adjusted image
